# Novel 4-Chromanone-Derived Compounds as Plant Immunity Inducers against CMV Disease in *Passiflora* spp. (Passion Fruit)

**DOI:** 10.3390/molecules29051045

**Published:** 2024-02-28

**Authors:** Tianli Wu, Lu Yu, Lingling Xiao, Tao Wang, Pei Li, Bo Mu

**Affiliations:** 1 School of Liquor and Food Engineering, Guizhou University, Guiyang 550025, China; w1612281228@163.com (T.W.); an1378386891@163.com (L.X.); 2 Guizhou Light Industry Technical College, Guiyang 550032, China; 3 Qiandongnan Engineering and Technology Research Center for Comprehensive Utilization of National Medicine, Kaili University, Kaili 556011, China; 4 Guizhou Academy of Testing and Analysis, Guiyang 550000, China; 18143522730@163.com

**Keywords:** 4-chromanone, plant immune inducer, *Passiflora* spp., anti-CMV activity, ABA signaling pathway

## Abstract

This study involved the design and synthesis of a series of novel 4-chromanone-derived compounds. Their in vivo anti-cucumber mosaic virus (CMV) activity in field trials against CMV disease in *Passiflora* spp. was then assessed. Bioassay results demonstrated that compounds **7c** and **7g** exhibited remarkable curative effects and protection against CMV, with inhibition rates of 57.69% and 51.73% and 56.13% and 52.39%, respectively, surpassing those of dufulin and comparable to ningnanmycin. Field trials results indicated that compound **7c** displayed significant efficacy against CMV disease in *Passiflora* spp. (passion fruit) after the third spraying at a concentration of 200 mg/L, with a relative control efficiency of 47.49%, surpassing that of dufulin and comparable to ningnanmycin. Meanwhile, nutritional quality test results revealed that compound **7c** effectively enhanced the disease resistance of *Passiflora* spp., as evidenced by significant increases in soluble protein, soluble sugar, total phenol, and chlorophyll contents in *Passiflora* spp. leaves as well as improved the flavor and taste of *Passiflora* spp. fruits, as demonstrated by notable increases in soluble protein, soluble sugar, soluble solid, and vitamin C contents in *Passiflora* spp. fruits. Additionally, a transcriptome analysis revealed that compound **7c** primarily targeted the abscisic acid (ABA) signaling pathway, a crucial plant hormone signal transduction pathway, thereby augmenting resistance against CMV disease in *Passiflora* spp. Therefore, this study demonstrates the potential application of these novel 4-chromanone-derived compounds as effective inducers of plant immunity for controlling CMV disease in *Passiflora* spp. in the coming decades.

## 1. Introduction

*Passiflora* spp. (passion fruit) is highly nutritious and possesses an exquisite sweet taste, making it widely cherished by the majority of consumers [1]. However, with the extensive cultivation of passion fruit in China, diseases have proliferated across all provinces due to the introduction, sale, and propagation of infected seedlings [2]. Among these diseases, cucumber mosaic virus (CMV) disease stands out as one of the primary viral infections affecting passion fruit plants [2,3]. This disease significantly diminishes both production and quality within the *Passiflora* spp. industry. To date, a wide variety of commercial antiviral agents targeting *Passiflora* spp. CMV disease have been reported on the market including chitosan oligosaccharide, dufulin, and ningnanmycin [4]. While traditional chemical antiviral agents have proven effective in treating *Passiflora* spp. CMV disease, their usage also raises numerous environmental concerns [5]. Therefore, there is an urgent need to focus on developing new innovative and promising antiviral agents for *Passiflora* spp. CMV disease in the coming decades.

**Figure 1:**
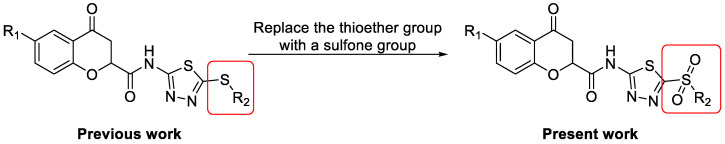
The design idea for the target compounds.

The utilization of natural product pesticides for plant disease management represents a pioneering approach toward achieving sustainable agricultural development in the 21st century and beyond [6]. Rich structures, target species specificity, unique modes of action, and biodegradability make natural products an inspiring source for the discovery of lead compounds in pesticides [7]. The exploration of novel active components and the development of new pesticides through structural modifications of natural products are crucial strategies [8]. Chromone, a botanical active component, and its derivatives are widely distributed in various plant parts such as roots, flowers, pericarps, and stems, exhibiting a broad spectrum of biological properties including antifungal, antibacterial, antiviral, and anticancer properties [9,10,11,12,13,14,15,16]. 4-Chromanone, which belongs to the chromone compound family, exhibits a wide range of significant biological and pharmaceutical activities, including anticancer, antioxidant, antifungal, and antibacterial activities [17]. Numerous studies have demonstrated that structural alterations in 4-chromanone, particularly at the 2 or 3 position, offer significant diversity which facilitates the development of novel active molecules [18,19,20,21]. Meanwhile, in our previous work, a series of novel 4-chromanone derivatives (Figure 1) were synthesized and employed for the management of plant bacterial and fungal diseases; our bioassay screening results indicated that the target compounds exhibited moderate-to-potent antibacterial activities against *Xanthomonas axonopodis* pv. *citri* and *Xanthomonas oryzae* pv. *oryzicolaby*; however, they demonstrated weaker inhibitory effects on *Mucor bainieri*, *Mucor fragilis*, and *Trichoderma atroviride* [22].

To further expand upon our previous investigation and develop novel lead compounds with enhanced bioactivity, the objective of this study was to substitute a thioether group with a sulfone group in order to synthesize novel 4-chromanone-derived compounds containing a sulfone moiety (Figure 1). Subsequently, these compounds were subjected to laboratory antiviral activity assays and field trial tests against *Passiflora* spp. CMV disease.

## 2. Results and Discussion

### 2.1. Chemistry

**Figure 2:**
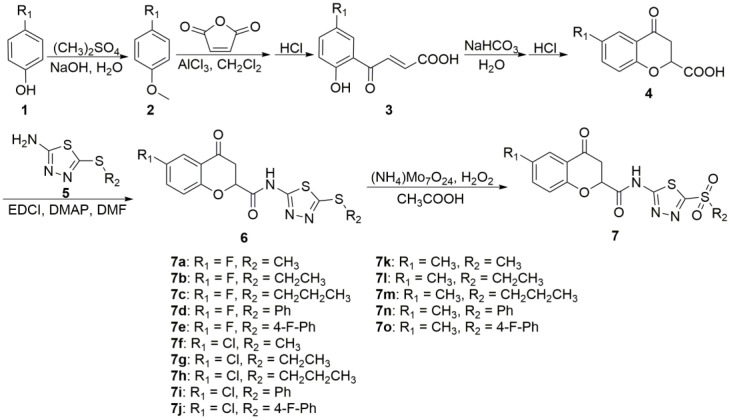
General protocols for the synthesis of target compounds **7a**–**7o**.

The synthesis of compounds **7a**–**7o**, as depicted in Figure 2, was accomplished through a five-step reaction route involving substitution, Michael addition, cyclization, condensation, and oxidation reactions, following established protocols [22,23]. The structures of the target compounds **7a**–**7o** were confirmed through analyses using ^1^H NMR, ^13^C NMR, and HRMS techniques. The representative data for the target compounds **7a**–**7o** are provided below. As an illustration, the integration of ^1^H NMR spectra confirms the presence of hydrogen atoms in compound **7c**, which is consistent with the proposed structure. A double-doublet (dd) peak at 5.59 ppm and two dd peaks at 3.22 and 3.16 ppm were associated with the presence of OCH and CH_2_ in the 4-chromanone group, respectively. Similar results were also reported in our previous study [22].

Data for 6-fluoro-*N*-(5-(methylsulfonyl)-1,3,4-thiadiazol-2-yl)-4-oxochromane-2-carboxamide (**7a**). Yellow solid, mp 226–228 °C, yield 52%; ^1^H NMR (400 MHz, DMSO-*d*_6_, ppm) *δ*: 13.81 (s, 1H, CONH), 7.54–7.45 (m, 2H, Ar-H), 7.26 (dd, *J* = 8.0, 4.0 Hz, 1H, Ar-H), 5.59 (dd, *J* = 8.0, 4.0 Hz, 1H, OCH), 3.56 (s, 3H, CH_3_), 3.22 (dd, *J* = 16.0, 4.0 Hz, 1H, CH_2_), 3.16 (dd, *J* = 16.0, 8.0 Hz, 1H, CH_2_); ^13^C NMR (100 MHz, DMSO-*d*_6_, ppm) *δ*: 189.4 (C=O), 168.6 (C=O), 163.1 (Ar-C–F, d, *J* = 102.0 Hz), 158.5 (Ar-C), 156.2 (Ar-C, d, *J* = 10.0 Hz), 124.3 (Ar-C, d, *J* = 25.0 Hz), 121.8 (Ar-C), 120.8 (Ar-C, d, *J* = 7.0 Hz), 111.6 (Ar-C), 111.4 (Ar-C), 76.0 (CH_2_), 43.8 (OCH), 38.5 (CH_3_); HRMS (ESI) [M + Na]^+^ calcd for C_13_H_10_FN_3_O_5_S_2_: 393.99381, found 393.99308.

Data for *N*-(5-(ethylsulfonyl)-1,3,4-thiadiazol-2-yl)-6-fluoro-4-oxochromane-2-carboxamide (**7b**). Yellow solid, mp 215–217 °C, yield 50%; ^1^H NMR (400 MHz, DMSO-*d*_6_, ppm) *δ*: 13.81 (s, 1H, CONH), 7.55–7.45 (m, 2H, Ar-H), 7.26 (dd, *J* = 8.0, 4.0 Hz, 1H, Ar-H), 5.59 (dd, *J* = 8.0, 4.0 Hz, 1H, OCH), 3.66 (q, *J* = 8.0, 4.0 Hz, 2H, CH_2_CH_3_), 3.22 (dd, *J* = 16.0, 4.0 Hz, 1H, CH_2_), 3.16 (dd, *J* = 16.0, 8.0 Hz, 1H, CH_2_), 1.25 (t, *J* = 4.0 Hz, 3H, CH_2_CH_3_); ^13^C NMR (100 MHz, DMSO-*d*_6_, ppm) *δ*: 189.4 (C=O), 168.6 (C=O), 162.4 (Ar-C-F, d, *J* = 88.0 Hz), 158.5 (Ar-C), 156.3 (Ar-C), 124.3 (Ar-C, d, *J* = 25.0 Hz), 121.7 (Ar-C, d, *J* = 6.0 Hz), 120.7 (Ar-C, d, *J* = 8.0 Hz), 111.6 (Ar-C), 111.4 (Ar-C), 76.0 (CH_2_), 50.2 (OCH), 38.5 (CH_2_), 7.4 (CH_3_); HRMS (ESI) [M + Na]^+^ calcd for C_14_H_12_FN_3_O_5_S_2_: 408.00946, found 408.00934.

Data for 6-fluoro-4-oxo-*N*-(5-(propylsulfonyl)-1,3,4-thiadiazol-2-yl)chromane-2-carboxamide (**7c**). Yellow solid, mp 220–221 °C, yield 47%; ^1^H NMR (400 MHz, DMSO-*d*_6_, ppm) *δ*: 13.81 (s, 1H, CONH), 7.55–7.45 (m, 2H, Ar-H), 7.26 (dd, *J* = 8.0, 4.0 Hz, 1H, Ar-H), 5.59 (dd, *J* = 8.0, 4.0 Hz, 1H, OCH), 3.64 (t, *J* = 8.0 Hz, 2H, CH_2_CH_2_CH_3_), 3.22 (dd, *J* = 16.0, 4.0 Hz, 1H, CH_2_), 3.16 (dd, *J* = 16.0, 8.0 Hz, 1H, CH_2_), 1.76–1.67 (m, 2H, CH_2_CH_2_CH_3_), 0.96 (t, *J* = 4.0 Hz, 3H, CH_2_CH_2_CH_3_); ^13^C NMR (100 MHz, DMSO-*d*_6_, ppm) *δ*: 189.4 (C=O), 168.6 (C=O), 162.8 (Ar-C), 162.4 (Ar-C), 158.5 (Ar-C), 156.3 (Ar-C), 156.2 (Ar-C, d, *J* = 13.0 Hz), 124.3 (Ar-C, d, *J* = 24.0 Hz), 121.7 (Ar-C), 120.7 (Ar-C), 111.6 (Ar-C), 111.4 (Ar-C), 76.0 (CH_2_), 56.8 (OCH), 38.5 (CH_2_), 16.5 (CH_2_), 12.9 (CH_3_); HRMS (ESI) [M + Na]^+^ calcd for C_15_H_14_FN_3_O_5_S_2_: 422.02511, found 422.02494.

Data for *N*-(5-(benzylsulfonyl)-1,3,4-thiadiazol-2-yl)-6-fluoro-4-oxochromane-2-carboxamide (**7d**). White solid, mp 245–246 °C, yield 60%; ^1^H NMR (400 MHz, DMSO-*d*_6_, ppm) *δ*: 13.78 (s, 1H, CONH), 7.53–7.45 (m, 2H, Ar-H), 7.35–7.34 (m, 3H, Ar-H), 7.29–7.23 (m, 3H, Ar-H), 5.56 (dd, *J* = 8.0, 4.0 Hz, 1H, OCH), 5.05 (s, 2H, SO_2_CH_2_), 3.19 (dd, *J* = 16.0, 4.0 Hz, 1H, CH_2_), 3.14 (dd, *J* = 16.0, 8.0 Hz, 1H, CH_2_); ^13^C NMR (100 MHz, DMSO-*d*_6_, ppm) *δ*: 189.4 (C=O), 170.4 (C=O), 168.6 (Ar-C), 162.4 (Ar-C-F, d, *J* = 123.0 Hz), 158.6 (Ar-C), 156.6 (Ar-C), 131.8 (Ar-C), 129.2 (Ar-C, d, *J* = 33.0 Hz), 127.5 (Ar-C), 124.3 (Ar-C, d, *J* = 25.0 Hz), 121.8 (Ar-C), 121.7 (Ar-C), 120.9 (Ar-C), 120.8 (Ar-C), 120.7 (Ar-C), 111.6 (Ar-C), 111.4 (Ar-C), 76.1 (CH_2_), 61.3 (OCH), 38.5 (CH_2_); HRMS (ESI) [M + Na]^+^ calcd for C_19_H_14_FN_3_O_5_S_2_: 470.02511, found 470.02449.

Data for 6-fluoro-*N*-(5-((4-fluorobenzyl)sulfonyl)-1,3,4-thiadiazol-2-yl)-4-oxochromane-2-carboxamide (**7e**). White solid, mp 254–256 °C, yield 40%; ^1^H NMR (400 MHz, DMSO-*d*_6_, ppm) *δ*: 13.79 (s, 1H, CONH), 7.53–7.45 (m, 2H, Ar-H), 7.35–7.32 (m, 2H, Ar-H), 7.27–7.17 (m, 3H, Ar-H), 5.57 (dd, *J* = 8.0, 4.0 Hz, 1H, OCH), 5.08 (s, 2H, SO_2_CH_2_), 3.22–3.12 (m, 2H, CH_2_); ^13^C NMR (100 MHz, DMSO-*d*_6_, ppm) *δ*: 189.4 (C=O), 168.6 (C=O), 162.4 (Ar-C-F, d, *J* = 139.0 Hz), 161.6 (Ar-C), 156.3 (Ar-C), 134.0 (Ar-C, d, *J* = 9.0 Hz), 124.4 (Ar-C, d, *J* = 25.0 Hz), 123.9 (Ar-C), 123.9 (Ar-C), 121.8 (Ar-C), 121.7 (Ar-C, d, *J* = 7.0 Hz), 120.7 (Ar-C, d, *J* = 7.0 Hz), 116.2 (Ar-C), 116.0 (Ar-C), 111.6 (Ar-C), 111.4 (Ar-C), 76.1 (CH_2_), 60.3 (OCH), 38.5 (CH_2_); HRMS (ESI) [M + Na]^+^ calcd for C_19_H_13_F_2_N_3_O_5_S_2_: 488.01569, found 488.01468.

Data for 6-chloro-*N*-(5-(methylsulfonyl)-1,3,4-thiadiazol-2-yl)-4-oxochromane-2-carboxamide (**7f**). Yellow solid, mp 266–268 °C, yield 62%; ^1^H NMR (400 MHz, DMSO-*d*_6_, ppm) *δ*: 13.82 (s, 1H, CONH), 7.70–7.66 (m, 2H, Ar-H), 7.26–7.23 (m, 1H, Ar-H), 5.62 (dd, *J* = 8.0, 4.0 Hz, 1H, OCH), 3.55 (s, 3H, CH_3_), 3.24 (dd, *J* = 16.0, 4.0 Hz, 1H, CH_2_), 3.17 (dd, *J* = 16.0, 8.0 Hz, 1H, CH_2_); ^13^C NMR (100 MHz, DMSO-*d*_6_, ppm) *δ*: 189.0 (C=O), 168.5 (C=O), 163.6 (Ar-C), 162.6 (Ar-C), 158.6 (Ar-C), 136.5 (Ar-C), 126.7 (Ar-C), 125.6 (Ar-C), 122.2 (Ar-C), 120.9 (Ar-C), 76.0 (CH_2_), 43.8 (OCH), 38.5 (CH_3_); HRMS (ESI) [M + Na]^+^ calcd for C_13_H_10_ClN_3_O_5_S_2_: 409.96426, found 409.96430.

Data for 6-chloro-*N*-(5-(ethylsulfonyl)-1,3,4-thiadiazol-2-yl)-4-oxochromane-2-carboxamide (**7g**). Yellow solid, mp 249–251 °C, yield 54%; ^1^H NMR (400 MHz, DMSO-*d*_6_, ppm) *δ*: 13.82 (s, 1H, CONH), 7.70–7.66 (m, 2H, Ar-H), 7.26–7.23 (m, 1H, Ar-H), 5.62 (dd, *J* = 8.0, 4.0 Hz, 1H, OCH), 3.66 (q, *J* = 16.0, 8.0 Hz, 2H, CH_2_CH_3_), 3.24 (dd, *J* = 16.0, 4.0 Hz, 1H, CH_2_), 3.17 (dd, *J* = 16.0, 8.0 Hz, 1H, CH_2_), 1.25 (t, *J* = 4.0 Hz, 3H, CH_2_CH_3_); ^13^C NMR (100 MHz, DMSO-*d*_6_, ppm) *δ*: 189.0 (C=O), 168.6 (C=O), 162.9 (Ar-C), 162.0 (Ar-C), 158.6 (Ar-C), 136.5 (Ar-C), 126.7 (Ar-C), 125.6 (Ar-C), 122.2 (Ar-C), 120.9 (Ar-C), 76.1 (CH_2_), 50.2 (OCH), 38.5 (CH_2_), 7.4 (CH_3_); HRMS (ESI) [M + Na]^+^ calcd for C_14_H_12_ClN_3_O_5_S_2_: 423.97991, found 423.97918.

Data for 6-chloro-4-oxo-*N*-(5-(propylsulfonyl)-1,3,4-thiadiazol-2-yl)chromane-2-carboxamide (**7h**). Yellow solid, mp 183–184 °C, yield 50%; ^1^H NMR (400 MHz, DMSO-*d*_6_, ppm) *δ*: 13.81 (s, 1H, CONH), 7.70–7.66 (m, 2H, Ar-H), 7.26–7.23 (m, 1H, Ar-H), 5.62 (dd, *J* = 8.0, 4.0 Hz, 1H, OCH), 3.63 (t, *J* = 8.0 Hz, 2H, CH_2_CH_2_CH_3_), 3.24 (dd, *J* = 16.0, 4.0 Hz, 1H, CH_2_), 3.17 (dd, *J* = 16.0, 8.0 Hz, 1H, CH_2_), 1.76–1.66 (m, 2H, CH_2_CH_2_CH_3_), 0.96 (t, *J* = 4.0 Hz, 3H, CH_2_CH_2_CH_3_); ^13^C NMR (100 MHz, DMSO-*d*_6_, ppm) *δ*: 189.0 (C=O), 168.5 (C=O), 162.8 (Ar-C), 162.5 (Ar-C), 158.6 (Ar-C), 136.5 (Ar-C), 126.7 (Ar-C), 125.6 (Ar-C), 122.2 (Ar-C), 120.9 (Ar-C), 76.0 (CH_2_), 56.8 (OCH), 38.5 (CH_2_), 16.5 (CH_2_), 12.9 (CH_3_); HRMS (ESI) [M + Na]^+^ calcd for C_15_H_14_ClN_3_O_5_S_2_: 437.99556, found 437.19301.

Data for *N*-(5-(benzylsulfonyl)-1,3,4-thiadiazol-2-yl)-6-chloro-4-oxochromane-2-carboxamide (**7i**). Yellow solid, mp 244–245 °C, yield 45%; ^1^H NMR (400 MHz, DMSO-*d*_6_, ppm) *δ*: 13.79 (s, 1H, CONH), 7.69–7.66 (m, 2H, Ar-H), 7.36–7.22 (m, 6H, Ar-H), 5.59 (dd, *J* = 8.0, 4.0 Hz, 1H, OCH), 5.05 (s, 2H, SO_2_CH_2_), 3.21 (dd, *J* = 16.0, 4.0 Hz, 1H, CH_2_), 3.15 (dd, *J* = 16.0, 8.0 Hz, 1H, CH_2_); ^13^C NMR (100 MHz, DMSO-*d*_6_, ppm) *δ*: 189.0 (C=O), 168.5 (C=O), 163.0 (Ar-C), 161.8 (Ar-C), 158.6 (Ar-C), 136.5 (Ar-C), 131.8 (Ar-C), 129.4 (Ar-C), 129.1 (Ar-C), 127.5 (Ar-C), 126.7 (Ar-C), 125.6 (Ar-C), 122.2 (Ar-C), 120.9 (Ar-C), 76.0 (CH_2_), 61.3 (OCH), 38.5 (CH_2_); HRMS (ESI) [M + Na]^+^ calcd for C_19_H_14_ClN_3_O_5_S_2_: 461.99906, found 461.99923.

Data for 6-chloro-*N*-(5-((4-fluorobenzyl)sulfonyl)-1,3,4-thiadiazol-2-yl)-4-oxochromane-2-carboxamide (**7j**). White solid, mp 256–257 °C, yield 40%; ^1^H NMR (400 MHz, DMSO-*d*_6_, ppm) *δ*: 13.80 (s, 1H, CONH), 7.69–7.66 (m, 2H, Ar-H), 7.35–7.32 (m, 2H, Ar-H), 7.26–7.17 (m, 3H, Ar-H), 5.60 (dd, *J* = 8.0, 4.0 Hz, 1H, CH), 5.08 (s, 2H, SO_2_CH_2_), 3.22 (dd, *J* = 16.0, 4.0 Hz, 1H, CH_2_), 3.16 (dd, *J* = 16.0, 8.0 Hz, 1H, CH_2_); ^13^C NMR (100 MHz, DMSO-*d*_6_, ppm) *δ*: 189.0 (C=O), 168.5 (C=O), 163.6 (Ar-C-F, d, *J* = 103 Hz), 161.6 (Ar-C), 158.6 (Ar-C), 136.5 (Ar-C), 134.0 (Ar-C, d, *J* = 9.0 Hz), 126.7 (Ar-C), 125.6 (Ar-C), 123.9 (Ar-C), 122.2 (Ar-C), 120.9 (Ar-C), 116.2 (Ar-C), 116.0 (Ar-C), 76.1 (CH_2_), 60.3 (OCH), 38.5 (CH_2_); HRMS (ESI) [M + Na]^+^ calcd for C_19_H_13_ClFN_3_O_5_S_2_: 503.98614, found 503.98554.

Data for 6-methyl-*N*-(5-(methylsulfonyl)-1,3,4-thiadiazol-2-yl)-4-oxochromane-2-carboxamide. (**7k**). White solid, mp 240–242 °C, yield 48%; ^1^H NMR (400 MHz, DMSO-*d*_6_, ppm) *δ*: 13.77 (s, 1H, CONH), 7.55 (d, *J* = 4.0 Hz, 1H, Ar-H), 7.44 (dd, *J* = 8.0 Hz, 4.0 Hz, 1H, Ar-H), 7.08 (d, *J* = 8.0 Hz, Ar-H), 5.54 (dd, *J* = 8.0 Hz, 4.0 Hz, 1H, CH), 3.55 (s, 3H, CH_3_), 3.16 (dd, *J* = 16.0, 4.0 Hz, 1H, CH_2_), 3.12 (dd, *J* = 16.0, 4.0 Hz, 1H, CH_2_), 2.28 (s, 3H, CH_3_); ^13^C NMR (100 MHz, DMSO-*d*_6_, ppm) *δ*: 190.0 (C=O), 168.9 (C=O), 163.5 (Ar-C), 162.6 (Ar-C), 157.9 (Ar-C), 137.8 (Ar-C), 131.7 (Ar-C), 126.2 (Ar-C), 120.8 (Ar-C), 118.3 (Ar-C), 75.9 (CH_2_), 43.8 (OCH), 38.8 (CH_3_), 20.4 (CH_3_); HRMS (ESI) [M + Na]^+^ calcd for C_14_H_13_N_3_O_5_S_2_: 390.018883, found 390.01893.

Data for *N*-(5-(ethylsulfonyl)-1,3,4-thiadiazol-2-yl)-6-methyl-4-oxochromane-2-carboxamide (**7l**). White solid, mp 206–207 °C, yield 51%; ^1^H NMR (400 MHz, DMSO-*d*_6_, ppm) *δ*: 13.77 (s, 1H, CONH), 7.55 (s, 1H, Ar-H), 7.44 (dd, *J* = 8.0, 4.0 Hz, 1H, Ar-H), 7.08 (d, *J* = 8.0 Hz, 1H, Ar-H), 5.53 (dd, *J* = 8.0, 4.0 Hz, 1H, OCH), 3.65 (q, *J* = 16.0 Hz, 8.0 Hz, 2H, CH_2_CH_3_), 3.16 (dd, *J* = 16.0, 4.0 Hz, 1H, CH_2_), 3.11 (dd, *J* = 16.0, 8.0 Hz, 1H, CH_2_), 2.28 (s, 3H, CH_3_), 1.24 (t, *J* = 4.0 Hz, 3H, CH_2_CH_3_); ^13^C NMR (100 MHz, DMSO-*d*_6_, ppm) *δ*: 190.0 (C=O), 168.9 (C=O), 162.9 (Ar-C), 161.9 (Ar-C), 158.0 (Ar-C), 137.8 (Ar-C), 131.7 (Ar-C), 126.2 (Ar-C), 120.8 (Ar-C), 118.3 (Ar-C), 75.9 (CH_2_), 50.2 (OCH), 38.8 (CH_2_), 20.4 (CH_3_), 7.4 (CH_3_); HRMS (ESI) [M + Na]^+^ calcd for C_15_H_15_N_3_O_5_S_2_: 404.03453, found 404.03410.

Data for 6-methyl-4-oxo-*N*-(5-(propylsulfonyl)-1,3,4-thiadiazol-2-yl)chromane-2-carboxamide (**7m**). White solid, mp 189–190 °C, yield 51%; ^1^H NMR (400 MHz, DMSO-*d*_6_, ppm) *δ*: 13.76 (s, 1H, CONH), 7.44 (d, *J* = 8.0 Hz, 1H, Ar-H), 7.08 (d, *J* = 8.0 Hz, 1H, Ar-H), 5.53 (dd, *J* = 8.0, 4.0 Hz, 1H, OCH), 3.63 (t, *J* = 8.0 Hz, 2H, CH_2_CH_2_CH_3_), 3.16 (dd, *J* = 16.0, 4.0 Hz, 1H, CH_2_), 3.11 (dd, *J* = 16.0, 8.0 Hz, 1H, CH_2_), 2.28 (s, 3H, CH_3_), 1.76–1.66 (m, 2H, CH_2_CH_2_CH_3_), 0.96 (t, *J* = 8.0 Hz, 3H, CH_2_CH_2_CH_3_); ^13^C NMR (100 MHz, DMSO-*d*_6_, ppm) *δ*: 190.0 (C=O), 168.9 (C=O), 162.9 (Ar-C), 162.4 (Ar-C), 158.0 (Ar-C), 137.8 (Ar-C), 131.7 (Ar-C), 126.2 (Ar-C), 120.8 (Ar-C), 118.3 (Ar-C), 75.9 (CH_2_), 56.8 (OCH), 38.8 (CH_2_), 20.4 (CH_3_), 16.5 (CH_2_), 12.9 (CH_3_); HRMS (ESI) [M + Na]^+^ calcd for C_16_H_17_N_3_O_5_S_2_: 418.05018, found 418.04983.

Data for *N*-(5-(benzylsulfonyl)-1,3,4-thiadiazol-2-yl)-6-methyl-4-oxochromane-2-carboxamide (**7n**). White solid, mp 233–235 °C, yield 41%; ^1^H NMR (400 MHz, DMSO-*d*_6_, ppm) *δ*: 13.75 (s, 1H, CONH), 7.55 (d, *J* = 4.0 Hz, 1H, Ar-H), 7.45 (dd, *J* = 8.0, 4.0 Hz, 1H, Ar-H), 7.35–7.26 (m, 5H, Ar-H), 7.08 (d, *J* = 8.0 Hz, 1H, Ar-H). 5.05 (s, 2H, SO_2_CH_2_), 3.16–3.06 (m, 2H, CH_2_), 2.28 (s, 3H, CH_3_); ^13^C NMR (100 MHz, DMSO-*d*_6_, ppm) *δ*: 190.0 (C=O), 168.8 (C=O), 163.1 (Ar-C), 161.7 (Ar-C), 157.9 (Ar-C), 137.8 (Ar-C), 131.8 (Ar-C), 129.4 (Ar-C), 129.1 (Ar-C), 127.5 (Ar-C), 126.2 (Ar-C), 120.8 (Ar-C), 118.3 (Ar-C), 75.9 (CH_2_), 61.3 (OCH), 38.8 (CH_2_), 20.4 (CH_3_); HRMS (ESI) [M + Na]^+^ calcd for C_20_H_17_N_3_O_5_S_2_: 466.05018, found 466.04975.

Data for *N*-(5-((4-fluorobenzyl)sulfonyl)-1,3,4-thiadiazol-2-yl)-6-methyl-4-oxochromane-2-carboxamide (**7o**). White solid, mp 235–237 °C, yield 41%; ^1^H NMR (400 MHz, DMSO-*d*_6_, ppm) *δ*: 13.77 (s, 1H, CONH), 7.55 (d, *J* = 4.0 Hz, 1H, Ar-H), 7.45 (dd, *J* = 8.0, 4.0 Hz, 1H, Ar-H), 7.35–7.31 (m, 2H, Ar-H), 7.23–7.17 (m, 2H, Ar-H), 7.08 (d, *J* = 8.0 Hz, 1H, Ar-H). 5.51 (dd, *J* = 8.0, 4.0 Hz, 1H, OCH), 5.07 (s, 2H, SO_2_CH_2_), 3.17–3.06 (m, 2H, CH_2_), 2.28 (s, 3H, CH_3_); ^13^C NMR (100 MHz, DMSO-*d*_6_, ppm) *δ*: 190.0 (C=O), 168.8 (C=O), 162.3 (Ar-C-F, d, *J* = 153.0 Hz), 157.9 (Ar-C), 137.8 (Ar-C), 134.0 (Ar-C, d, *J* = 9.0 Hz), 131.7 (Ar-C), 126.2 (Ar-C), 123.9 (Ar-C), 120.8 (Ar-C), 118.3 (Ar-C), 116.2 (Ar-C), 116.0 (Ar-C), 75.9 (CH_2_), 60.3 (OCH), 38.8 (CH_2_), 20.4 (CH_3_); HRMS (ESI) [M + Na]^+^ calcd for C_20_H_16_FN_3_O_5_S_2_: 484.04076, found 484.04051.

### 2.2. Results of Anti-CMV Activity and Field Trial Tests

In this study, the anti-CMV activity of target compounds **7a**–**7o** was evaluated at a concentration of 500 mg/L, and the results are recorded in Table 1. The data presented in Table 1 demonstrate that these compounds exhibited moderate to good protection and curative effects against CMV. Notably, compounds **7c** and **7g** exhibited remarkable protection activity against CMV, with inhibition rates of 57.69% and 56.13%, respectively, surpassing those of dufulin (42.08%) and ningnanmycin (50.13%). Moreover, compounds **7c** and **7g** demonstrated excellent curative activity against CMV, with inhibition rates of 51.73% and 52.39%, respectively, surpassing those of dufulin (40.78%) and comparable to ningnanmycin (51.35%). A structure–activity relationship (SAR) analysis showed that when R_1_ was a –F group, it was observed that the compound with a –CH_2_CH_2_CH_3_ group at the R_2_ substituent group exhibited favorable in vivo protection and curative activities in the following specific orders: **7c** > **7a** and **7c** > **7b**; when R_1_ was a –Cl group, the compound with a –CH_2_CH_3_ group at the R_2_ substituent group showed favorable in vivo protection and curative activities in the following specific orders: **7g** > **7f** and **7g** > **7h**.

In order to assess the control efficiency of compounds **7c** and **7g** against *Passiflora* spp. CMV disease, a preliminary field efficacy study was conducted at a concentration of 200 mg/L. According to the results presented in Table 2, after the first and second sprayings, both compounds **7c** and **7g** demonstrated effectiveness in reducing *Passiflora* spp. CMV disease at a concentration of 200 mg/L, exhibiting comparable relative control efficiencies to those of dufulin and ningnanmycin. Furthermore, after the third spraying, compound **7c** exhibited significant efficacy with a relative control efficiency of 47.49% at a concentration of 200 mg/L, surpassing that of dufulin (28.03%) and comparable to ningnanmycin (44.17%). However, following the third spraying, compound **7g** demonstrated significant efficacy with a relative control efficiency of 34.82% at a concentration of 200 mg/L, surpassing that of dufulin (28.03%) and falling below that of ningnanmycin (44.17%).

### 2.3. Results of Nutritional Quality of Passiflora spp. Leaves and Fruits

**Figure 3:**
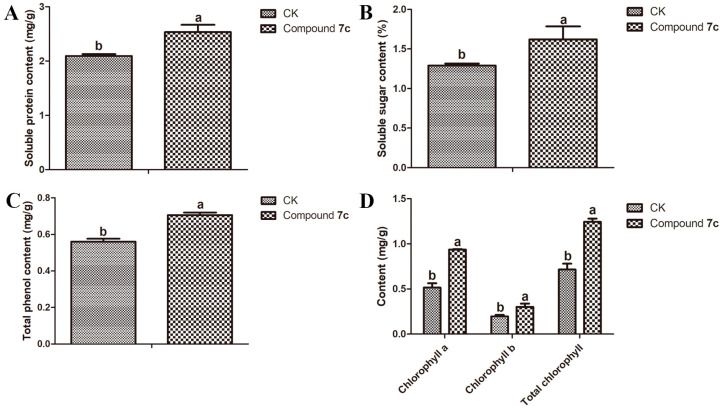
Effect of compound **7c** on the soluble protein (**A**), soluble sugar (**B**), total phenol (**C**), and chlorophyll (**D**) contents of *Passiflora* spp. leaves. The nutritional quality of *Passiflora* spp. leaves was significantly different among various lowercase letters at a significance level of *p* < 0.05. Error bars refer to mean ± SD (*n* = 3) values.

The presence of soluble proteins in plants is essential for enhancing cellular water retention, providing cellular protection, and bolstering plant disease resistance [24]. The role of soluble sugars in the life cycle of plants is pivotal as they not only serve as a primary source of energy and metabolic intermediates for plant growth and development but also play a crucial role as signaling molecules [25]. Phenol compounds are classified as polyhydroxyl secondary metabolites that exhibit notable antioxidant activity and have been found to be closely associated with enhancing plant resistance against diseases [26]. The content of chlorophyll, the photosynthetic pigment in green plants, is intricately associated with photosynthesis and proliferation, as well as factors that hinder chlorophyll synthesis, resulting in leaf chlorosis to enhance photosynthesis and strengthen plant defense against microbial infections [27]. The results depicted in Figure 3 demonstrate that compound **7c** could effectively enhance resistance against *Passiflora* spp. CMV disease by significantly increasing the soluble protein, soluble sugar, total phenol, and chlorophyll contents.

**Figure 4:**
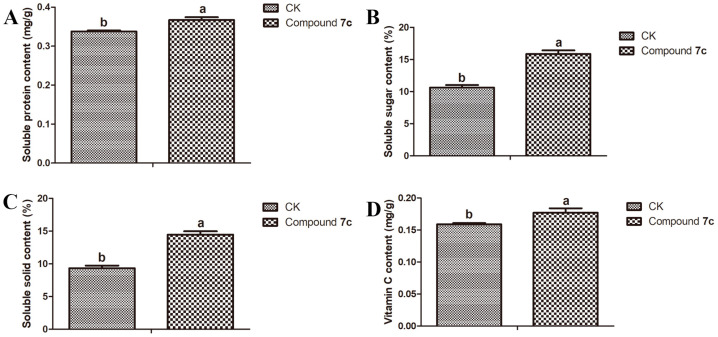
Effect of compound **7c** on the soluble protein (**A**), soluble sugar (**B**), soluble solid (**C**), and vitamin C (**D**) contents of *Passiflora* spp. fruits. The nutritional quality of *Passiflora* spp. fruits was significantly different among various lowercase letters at a significance level of *p* < 0.05. Error bars refer to mean ± SD (*n* = 3) values.

In recent years, with the growing emphasis on personal health, consumers have increasingly demanded fruits with higher nutritional quality. It is widely acknowledged that key indicators of nutritional quality, such as soluble protein, soluble sugar, soluble solid, and vitamin C contents, play a pivotal role in determining the flavor and taste of fruits while also influencing consumer purchasing decisions [28]. Soluble protein is of paramount importance to fruit growers as it plays a crucial role in enhancing the flavor and taste of fruits, thereby stimulating consumer purchases [29]. Soluble sugar, as one of the primary determinants influencing fruit quality, exerts a direct impact on the sweetness of fresh fruits while also serving as a precursor for synthesizing other compounds related to quality, including organic acids, anthocyanins, and aroma compounds [30]. Soluble solids, a crucial parameter in the fruit ripening process and economic benefits, are closely related to fruit flavor and optimal harvest time [31]. The nutritional component vitamin C is a paramount vitamin for human nutrition that is sourced from fruits [32]. The results depicted in Figure 4 demonstrate that treatment with compound **7c** significantly enhances the contents of soluble protein, soluble sugar, soluble solid, and vitamin C in *Passiflora* spp. fruits, which not only improves the nutritional quality but also enhances the flavor and taste of *Passiflora* spp. fruits.

### 2.4. Quality Check of Transcriptome Sequencing Data

The cDNA libraries of *Passiflora* spp. leaves treated with compound **7c** and the CK group were analyzed using the Illumina HiSeq platform to investigate gene expression information at the transcriptome level [33]. The transcriptome sequencing data were evaluated for a valid ratio, Q20 and Q30 base proportions, and GC content, with summarized results presented in Table 3. Based on the findings from Table 3, it can be concluded that the sequencing of both samples exhibited exceptional quality, with valid ratios > 98%, proportions of Q20 bases > 98%, proportions of Q30 bases > 93%, and GC contents > 43%, indicating suitability for a subsequent bioinformatics analysis [34].

### 2.5. Results of DEG and Bioinformatic Analyses

**Figure 5:**
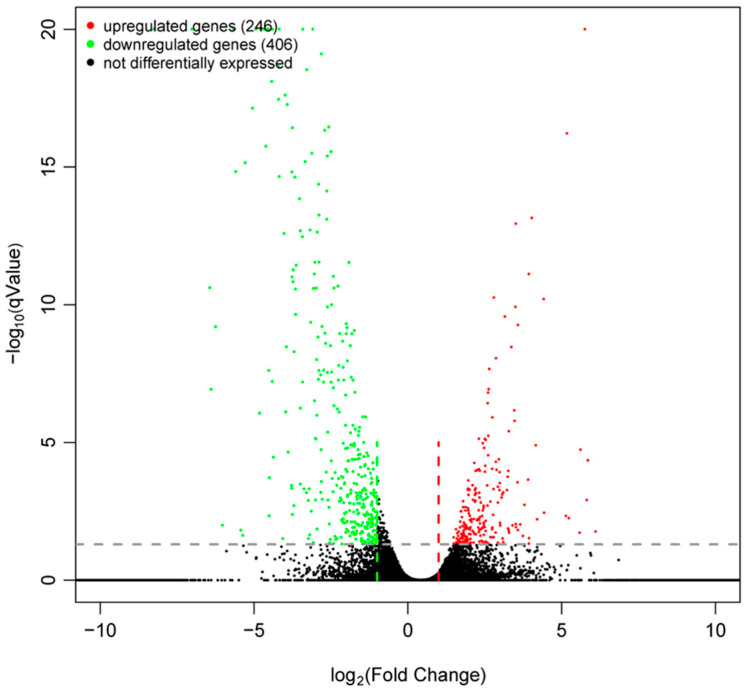
Volcanic map of DEGs.

The results, as depicted in Figure 5 and Table S1, demonstrate that a total of 652 DEGs were identified, with 246 upregulated and 406 downregulated genes observed in the compound **7c** treatment group compared to the CK group.

**Figure 6:**
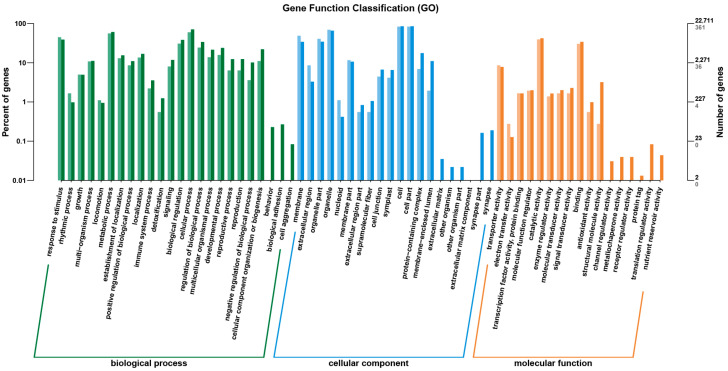
GO classification of DEGs.

Meanwhile, a functional annotation of these DEGs was performed using a GO analysis to gain insights into their biological significance. As depicted in Figure 6 and Table S2, the GO analysis revealed the enrichment of 150 GO functions by the DEGs, encompassing 77 BP (cellular process (GO:0009987), metabolic process (GO:0008152), biological regulation (GO:0065007), the regulation of biological process (GO:0050789), response to stimulus (GO:0050896), etc.), 43 CC (cell (GO:0005623), cell part (GO:0044464), organelle (GO:0043226), membrane (GO:0016020), organelle part (GO:0044422). etc.), and 30 MF (binding (GO:0005488), catalytic activity (GO:0003824), transporter activity (GO:0005215), molecular function regulator (GO:0000988), signal transducer activity (GO:0004871), etc.) categories.

**Figure 7:**
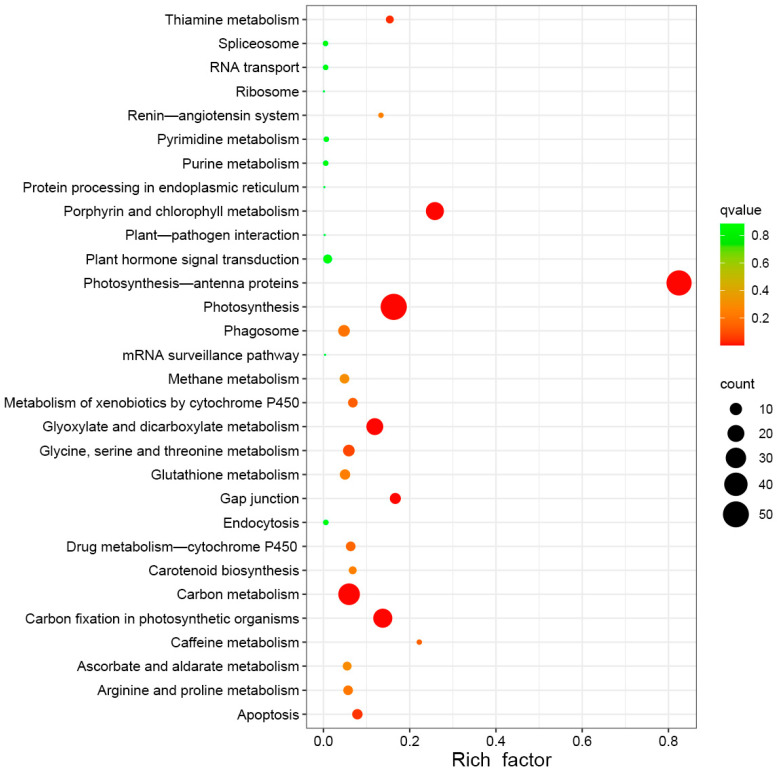
Top thirty KEGG pathway enrichments of DEGs.

In addition, Figure 7 and Table S3 show that the enriched KEGG pathways of the DEGs were photosynthesis (ko00195), photosynthesis antenna proteins (ko00196), porphyrin and chlorophyll metabolism (ko00860), carbon fixation in photosynthetic organisms (ko00710), glyoxylate and dicarboxylate metabolism (ko00630), carbon metabolism (ko01200), gap junction (ko04540), apoptosis (ko04210), glycine, serine, and threonine metabolism (ko00260), the metabolism of xenobiotics by cytochrome P450 (ko00980), drug metabolism—cytochrome P450 (ko00982), phagosome (ko04145), arginine and proline metabolism (ko00330), glutathione metabolism (ko00480), methane metabolism (ko00680), plant hormone signal transduction (ko04075), ascorbate and aldarate metabolism (ko00053), carotenoid biosynthesis (ko00906), etc.

**Figure 8:**
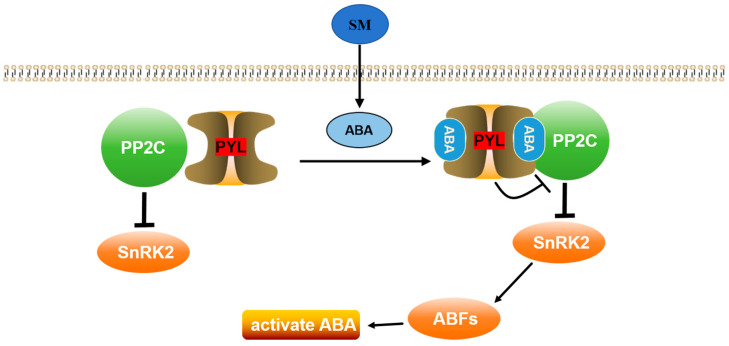
A model of the ABA signal transduction pathway in *Passiflora* spp. resistance to CMV disease. Supplementary Materials, compound **7c**.

In recent years, it has become evident that the interplay among various plant hormones regulates both plant growth and disease resistance. Additionally, plant hormone signal transduction may also play a role in the mechanisms underlying diverse pesticide applications and disease control techniques employed for crop protection [35]. The abscisic acid (ABA) signaling pathway is a pivotal plant hormone that regulates the response to abiotic stress and significantly impacts plants’ ability to defend against various pathogens [36]. The PYL and PP2C proteins, which are essential components of the ABA receptor-coupled core signaling pathway, were found to be significantly enriched in the plant hormone signal transduction pathway and were specifically annotated within the ABA signaling pathway [37]. In this study, a transcriptome analysis revealed that compound **7c** elicited an upregulation of *PYL* gene expression and a downregulation of *PP2C* gene expression. Therefore, it is speculated that 4-chromanone-derived compounds could mediate the ABA signaling pathway, thereby enhancing resistance to *Passiflora* spp. CMV disease (Figure 8). Numerous studies have demonstrated that when external stress triggers the production or release of ABA in plants, ABA binds to the PYR/PYL receptor, forming a complex that inhibits PP2C to facilitate the dissociation of SnRK2 from the PP2C complex, allowing it to phosphorylate downstream transcription factors and activate the ABA signaling pathway [38,39,40].

## 3. Materials and Methods

### 3.1. Chemical Synthesis

#### 3.1.1. General Protocols for the Synthesis of Intermediates **2**–**6**

Intermediates **2**–**6** were prepared using 4-substituted phenol as the starting material, following the established methods described in Figure 2 [22].

#### 3.1.2. General Protocols for the Synthesis of Target Compounds **7a**–**7o**

As depicted in Figure 2, the target compounds **7a**–**7o** were prepared according to the reported method [23]. Intermediate 6 (0.01 mol), acetic acid (10 mL), and ammonium molybdate (0.5 mmol), dissolved in 30% H_2_O_2_ (0.03 mol), were mixed in a 25 mL round-bottomed flask, followed by a reaction at room temperature for 1–4 h. Subsequently, the resulting mixture was poured into 50 mL of distilled water. Following filtration, the crude products were subjected to recrystallization with ethanol in order to obtain the pure target compounds **7a**−**7o**.

### 3.2. In Vivo Anti-CMV Activity Test

The curative activities and protection of the target compounds **7a**–**7o** against CMV in *Chenopodium amaranticolor* (*C. amaranticolor*) plants were assessed using the half-leaf method at a concentration of 500 mg/L, and the inhibition rate I (%) of the anti-CMV activity was calculated using the established method [41].

(1) $$\mathrm{Inhibition}\;\mathrm{rate}\;I\;(\%)=\frac{\mathrm{Average}\mathrm{local}\mathrm{lesion}\mathrm{number}\mathrm{of}\mathrm{control}\mathrm{group}-\mathrm{Average}\mathrm{local}\mathrm{lesion}\mathrm{number}\mathrm{smeared}\mathrm{with}\mathrm{compounds}}{\mathrm{Average}\mathrm{local}\mathrm{lesion}\mathrm{number}\mathrm{of}\mathrm{control}}\times 100\%$$

#### 3.2.1. The Curative Activities of the Title Compounds against CMV In Vivo

Leaves were selected from plants of the same age belonging to *C. amaranticolor* species. Subsequently, whole leaves were inoculated with crude CMV virus at a concentration of 6 × 10^−3^ mg/mL on both sides after previously treatment with silicon carbide. After 0.5 h, the leaves were washed and dried. Then, the target compound solutions were applied to the left side of the leaves while solvents were applied to the right side as a control measure. The number of local lesions was recorded 3 to 4 days post inoculation. Three replications were conducted for each compound in order to ensure result reliability.

#### 3.2.2. The Protective Activities of the Title Compounds against CMV In Vivo

Leaves of *C. amaranticolor* plants of the same age were selected. The compound solutions were applied to the left side of the leaves, while solvents were applied to the right side as a control. After 12 h, crude CMV (concentration of 6 × 10^−3^ mg/mL) was inoculated on both sides of the leaves at equal concentrations after prior scattering with silicon carbide. Following a 0.5 h interval, the leaves were rinsed with water and subsequently dried. The number of localized lesions was recorded between 3 and 4 days post inoculation. Each experiment involving each compound was conducted in triplicate to ensure result reliability.

### 3.3. Field Trials Test against Passiflora spp. CMV Disease

In 2023, field trials were conducted in Zhenning City (Guizhou Province, China) to evaluate the efficacy of compounds **7c** and **7g** against *Passiflora* spp. CMV disease at a concentration of 200 mg/L. Sterile distilled water was used as the CK group, while dufulin and ningnanmycin were employed as positive controls at the same concentration. Each group was set up with 4 plots, and the 5 groups were set up with a total of 20 plots, each of which was 15 m^2^, and the total land area was 300 m^2^. A total of three sprayings were conducted, with an interval of seven days between each spraying. The incidence in the community was assessed prior to spraying and then on the seventh day after each spraying. Three plants were randomly selected for investigation, and approximately 15 leaves were observed per plant. The disease index of the leaves corresponding to each disease grade was recorded based on the grading standard for diseased leaves. The following formula was used to calculate the control efficiencies of compounds **7c** and **7g** as well as dufulin and ningnanmycin against *Passiflora* spp. CMV disease on the 7th day after each of the three sprayings [42]. In the equation, C is the disease index of the CK group and T is the disease index of the treatment group. Each experiment was performed in triplicate to ensure result reliability.

(2) $$\mathrm{Control}\mathrm{efficiency}\;I\;(\%)=\frac{C-T}{C}\times 100\%$$

### 3.4. Nutritional Quality Test of Passiflora spp. Leaves and Fruits

To investigate the impact of compound **7c** on the nutritional composition of *Passiflora* spp. leaves, we determined the levels of soluble protein, soluble sugar, total phenol, and chlorophyll in *Passiflora* spp. leaves collected on the 7th day after the third spraying, using established methods [43,44,45,46]. Additionally, we conducted an analysis of the levels of soluble protein, soluble sugar, soluble solid, and vitamin C in *Passiflora* spp. fruits collected on the 7th day after the third spraying, following established protocols [43,44,47,48]. Each experiment was conducted in triplicate to ensure result reliability.

#### 3.4.1. Soluble Protein Test of *Passiflora* spp. Leaves and Fruits

To accurately measure a specific quantity (0.1 g) of *Passiflora* spp. leaves or fruits in triplicate, pulverize them thoroughly with liquid nitrogen and transfer the ground material into a test tube. Add a precise volume of distilled water to the test tube and centrifuge it at 12,000 rpm and 4 °C for 10 min to collect the supernatant as the protein extract. Take an appropriate amount of the extract and mix it with 5 mL of Coomassie Brilliant Blue solution on a vortex oscillator for a complete reaction over a period of 2 min. Measure the absorbance at 595 nm to calculate the soluble protein content using a standard curve generated using bovine serum protein.

#### 3.4.2. Soluble Sugar Test of *Passiflora* spp. Leaves and Fruits

The *Passiflora* spp. leaves or fruits (0.1 g) were accurately weighed in triplicate and fully ground with a small amount of liquid nitrogen in a mortar. Subsequently, 2.5 mL of distilled water was added, followed by boiling and filtration to obtain the soluble sugar extract. A certain volume of the extract was transferred into a test tube, along with distilled water, an anthranone–ethyl acetate mixed solution, and concentrated sulfuric acid. The mixture was then boiled in water for 1 min and cooled to room temperature, and the absorbance at 630 nm was measured. Finally, the content of soluble sugar was determined using a standard curve constructed from a sucrose standard solution.

#### 3.4.3. Chlorophyll Test of *Passiflora* spp. Leaves

The leaves of *Passiflora* spp. (0.1 g) were collected in triplicate for each treatment and then sliced into small, uniform pieces using a hole puncher, carefully avoiding the midrib. Subsequently, 50 mg samples were placed in 5 mL of a cold solution consisting of a 2:1 mixture of 85% acetone and 85% ethanol (*v*/*v*). These samples were homogenized, incubated at 35 °C for 0.5 h, and finally centrifuged at 6500 rpm for 15 min. Absorbance spectra were recorded at wavelengths of 663 and 645 nm to determine chlorophyll a and chlorophyll b levels, respectively, with reference to a standard solution. Chlorophyll a, chlorophyll b, and total chlorophyll contents were calculated using established methods.

#### 3.4.4. Total Phenol Test of *Passiflora* spp. Leaves

The *Passiflora* spp. leaves (0.1 g) were accurately weighed in triplicate and added to 2.5 mL of a hydrochloric acid–methanol solution for extraction, which lasted for 20 min. After centrifugation at 8000 rpm for 5 min, the resulting supernatant was collected as the total phenol extract. A certain amount of this extract was transferred into a 15 mL test tube, followed by the addition of 1 mL of a Folin–phenol reagent (diluted) and 2 mL of a sodium carbonate solution. The reaction occurred at room temperature for 30 min. Subsequently, the absorbance value was measured at a wavelength of 760 nm, and the content of total phenols was determined using a standard curve constructed with gallic acid.

#### 3.4.5. Vitamin C Test of *Passiflora* spp. Fruits

*Passiflora* spp. fruit was accurately weighed (0.1 g) in triplicate and then finely ground in a mortar using an oxalate–EDTA solution. The resulting mixture was carefully transferred into a 100 mL volumetric bottle and filtered to remove any impurities, and a specific amount of passion fruit extract was absorbed. Subsequently, 1mL of metaphosphate–acetic acid solution and a 5% sulfuric acid solution were added to the extract, followed by thorough mixing. Finally, 4 mL of ammonium molybdate solution was added to achieve a constant volume of 50 mL. The absorbance at a 705 nm wavelength was measured, and the vitamin C content was calculated using a standard curve obtained from an ascorbic acid standard solution.

#### 3.4.6. Soluble Solid Test of *Passiflora* spp. Fruits

The *Passiflora* spp. fruits (0.1 g) should be accurately selected for a specific quality, followed by homogenizing the pulp using a homogenizer. The resulting mixture should then be filtered through gauze and analyzed for soluble solids using a handheld refractometer (PAL-BXIACID F5, ATAGO, Tokyo, Japan).

### 3.5. Transcriptome Sequencing

The leaves of *Passiflora* spp. (15 leaves per plant) were collected and stored in a foam box with liquid nitrogen before they were subjected to transcriptome sequencing. The transcriptome was sequenced by Hangzhou Lianchuan Biological Co., LTD (Hangzhou, China), using the Illumina HiSeq™ 2000 platform (Illumina Inc., San Diego, CA, USA). The raw transcriptome sequencing data have been deposited in the National Center for Biotechnology Information (NCBI) database under the project ID PRJNA1061236. To obtain high-quality reads, Cutadapt software (version: cutadapt-1.9.3) was employed to delete undetermined and low-quality bases [49]. Gene expression levels were determined based on mean FPKM values: FPKM < 1 indicated non-expression; 1 ≤ FPKM < 10 indicated low expression; and FPKM ≥ 10 indicated high expression [50]. Differentially expressed genes (DEGs) were identified using an R language package at a significance level of adjusted *p* < 0.05 and −log_2_FC > 1 [51]. Gene ontology (GO), including cellular components (CCs), biological processes (BPs), and molecular function (MF), as well as Kyoto Encyclopedia of Genes and Genomes (KEGG) annotations for DEGs, were obtained from the GO database (http://www.geneontology.org/, 23 May 2023) and KEGG pathway database (http://www.genome.jp/Pathway, 23 May 2023) [4,52].

### 3.6. Statistical Analysis

The nutritional compositions of the *Passiflora* spp. leaves and fruits were subjected to a statistical analysis using a one-way analysis of variance (ANOVA), followed by the least significance difference test (LSD) in GraphPad Prism 5 (GraphPad Software Inc., San Diego, CA, USA).

## 4. Conclusions

In conclusion, our study findings demonstrate that compound **7c** effectively controls *Passiflora* spp. CMV disease and has the potential to be utilized as a plant immune inducer by primarily targeting the ABA signaling pathway to enhance resistance against *Passiflora* spp. CMV disease. Therefore, our study provides a theoretical basis for the utilization of 4-chromanone-derived compounds as plant immune inducers against *Passiflora* spp. CMV disease.

## Figures and Tables

**Table 1:** In vivo protection and curative activities of the target compounds **7a**–**7o** against CMV at a concentration of 500 mg/L.

Compounds	Inhibition Rate (Mean ± SD, %) *	
	Protection Activity	Curative Activity
**7a**	46.15 ± 2.28	30.84 ± 3.10
**7b**	31.17 ± 9.32	19.36 ± 2.83
**7c**	57.69 ± 5.06	51.73 ± 2.64
**7d**	26.28 ± 1.14	28.47 ± 4.47
**7e**	26.28 ± 1.14	30.61 ± 8.61
**7f**	27.11 ± 0.83	35.63 ± 6.52
**7g**	56.13 ± 3.59	52.39 ± 3.38
**7h**	39.34 ± 3.32	33.23 ± 5.44
**7i**	26.13 ± 3.36	22.56 ± 6.36
**7j**	25.26 ± 2.88	21.00 ± 7.56
**7k**	42.15 ± 1.56	39.56 ± 3.15
**7l**	29.20 ± 9.98	21.50 ± 6.83
**7m**	27.26 ± 3.16	32.97 ± 2.87
**7n**	22.17 ± 4.33	28.61 ± 4.32
**7o**	24.25 ± 4.31	16.23 ± 2.07
Dufulin	42.08 ± 6.28	40.78 ± 5.03
Ningnanmycin	50.13 ± 1.88	51.35 ± 1.95

* The experiments were conducted in triplicate.

**Table 2:** Field trials of compounds **7c** and **7g** against *Passiflora* spp. CMV disease at a concentration of 200 mg/L.

Compounds	Concentration (mg/L)	Control Efficiency (Mean ± SD, %) *		
		7 Days after the First Spraying	7 Days after the Second Spraying	7 Days after the Third Spraying
**7c**	200	28.88 ± 6.63 a	47.50 ± 8.98 a	47.49 ± 6.22 a
**7g**	200	30.63 ± 10.77 a	47.41 ± 9.40 a	34.82 ± 7.60 c
Dufulin	200	40.27 ± 17.05 a	42.90 ± 5.43 a	28.03 ± 1.92 c
Ningnanmycin	200	34.77 ± 3.15 a	40.39 ± 7.35 a	44.17 ± 4.14 a

* The relative control efficiency was significantly different among the different lowercase letters at a significance level of *p* < 0.05.

**Table 3:** A summary of the quality statistics results of the transcriptome sequencing data.

Treatment	Raw Data	Valid Data	Valid Ratio (%)	GC (%)	Q30 (%)	Q20 (%)
CK	38,186,288	37,866,831	99.16	44.78	93.40	98.02
Compound **7c** reatment	42,997,459	42,564,302	98.99	43.89	93.36	98.01

## Data Availability

Data are contained within the article and Supplementary Materials.

## Supplementary Materials

The following supporting information can be downloaded at https://www.mdpi.com/article/10.3390/molecules29051045/s1, Table S1: All the identified EDGs; Table S2: All the GO classifications of the DEGs; Table S3. All the KEGG pathway enrichments of the DEGs.

## References

[1] Corrêa R.C.G., Peralta R.M., Haminiuk C.W.I., Maciel G.M., Bracht A., Ferreira I.C.F.R. (2016). The past decade findings related with nutritional composition, bioactive molecules and biotechnological applications of *Passiflora* spp. (*Passion fruit*). Trends Food Sci. Technol..

[2] Chen L.J., Sun D.L., Zhang X.X., Shao D.Q., Lu Y.L., An Y.X. (2021). Transcriptome analysis of yellow passion fruit in response to cucumber mosaic virus infection. PLoS ONE.

[3] Huang A.J., Gu P.P., Wang Y., Li J.L., Yang Z.X., Yi L. (2023). Simultaneous detection and differentiation of four viruses in passion fruit plants by a multiplex RT-PCR. Trop. Plant Pathol..

[4] Zhang L.Q., Yu L., Zhao Z., Li P., Tan S.M. (2023). Chitosan oligosaccharide as a plant immune inducer on the *Passiflora* spp. (*Passion fruit*) CMV disease. Front. Plant Sci..

[5] Song B.A., Yang S., Jin L.H., Bhadury P.S. (2011). Environment-Friendly Antiviral Agents for Plants.

[6] Cantrell C.L., Dayan F.E., Duke S.O. (2012). Natural products as sources for new pesticides. J. Nat. Prod..

[7] Sparks T.C., Duke S.O. (2021). Structure simplification of natural products as a lead generation approach in agrochemical discovery. J. Agric. Food Chem..

[8] Gao B., Yang B., Feng X.D., Li C. (2022). Recent advances in the biosynthesis strategies of nitrogen heterocyclic natural products. Nat. Prod. Rep..

[9] Saengchantara S.T., Wallace T.W. (1986). Chromanols, chromanones, and chromones. Nat. Prod. Rep..

[10] Abdel Ghani S.B., Mugisha P.J., Wilcox J.C., Gado E.A.M., Medu E.O., Lamb A.J., Brown R.C.D. (2013). Convenient one-pot synthesis of chromone derivatives and their antifungal and antibacterial evaluation. Synth. Commun..

[11] Malefo M.S., Ramadwa T.E., Famuyide I.M., McGaw L.J., Eloff J.N., Sonopo M.S., Selepe M.A. (2020). Synthesis and antifungal activity of chromones and benzoxepines from the leaves of *Ptaeroxylon obliquum*. J. Nat. Prod..

[12] Jo H., Seo S.H., Na Y., Kwon Y. (2019). The synthesis and anticancer activities of chiral epoxy-substituted chromone analogs. Bioorg. Chem..

[13] Li M., Zan N.N., Huang M.X., Jiang D.H., Hu D.Y., Song B.A. (2020). Design, synthesis and anti-TMV activities of novel chromone derivatives containing dithioacetal moiety. Bioorg. Med. Chem. Lett..

[14] Shen S.Y., Xiong W., Li S.S., Liu X.S., Li Y.K., Miao D., Li X.M., Wang W.G., Du G., Gong D.P. (2023). Chromones from the tobacco derived fungus *Aspergillus versicolor* and their antiviral activity. Chem. Nat. Compd..

[15] Wu Y.P., Zhao G.K., Liu Z.Y., Tan T., Li Z.M., Zhou M., Yao H., Li Y.K., Wang W.G., Hu Q.F. (2023). Antiviral Chromone alkaloids from the cigar tobacco leaves derived endophytic fungus *Aspergillus lentulus*. Chem. Nat. Compd..

[16] Jiang D.H., Chen J.X., Zan N.N., Li C.Y., Hu D.Y., Song B.A. (2021). Discovery of novel chromone derivatives containing a sulfonamide moiety as anti-ToCV agents through the tomato chlorosis virus coat protein-oriented screening method. J. Agric. Food Chem..

[17] Diana E.J., Kanchana U.S., Mathew T.V. (2021). Current developments in the synthesis of 4-chromanone-derived compounds. Org. Biomol. Chem..

[18] Chouchéne N., Toumi A., Boudriga S., Edziri H., Sobeh M., Abdelfattah M.A.O., Askri M., Knorr M., Strohmann C., Brieger L. (2022). Antimicrobial activity and DFT studies of a novel set of spiropyrrolidines tethered with thiochroman-4-one/chroman-4-one scaffolds. Molecules.

[19] Ferreira A.R., Alves D.N., de Castro R.D., Perez-Castillo Y., de Sousa D.P. (2022). Synthesis of coumarin and homoisoflavonoid derivatives and analogs: The search for new antifungal agents. Pharmaceuticals.

[20] Kamboj S., Singh R. (2022). Chromanone-a prerogative therapeutic scaffold: An overview. Arab. J. Sci. Eng..

[21] Yang J., Lai J.X., Kong W.L., Li S.K. (2022). Asymmetric synthesis of sakuranetin-relevant flavanones for the identification of new chiral antifungal leads. J. Agric. Food Chem..

[22] Li J., Yu L., Xiao L.L., Yang M.W., Wu T.L., Zhang L.Q., Tan S.M., Li P. (2023). Design, synthesis, antibacterial, and antifungal evaluation of novel 4-chromanone-derived compounds incorporating carboxamide and 1,3,4-thiadiazole thioether moieties. Phosphorus Sulfur..

[23] Li P., Hu D.Y., Xie D.D., Chen J.X., Jin L.H., Song B.A. (2018). Design, synthesis, and evaluation of new sulfone derivatives containing a 1,3,4-oxadiazole moiety as active antibacterial agents. J. Agric. Food Chem..

[24] Tiwari R.K., Lal M.K., Kumar R., Mangal V., Altaf M.A., Sharma S., Singh B., Kumar M. (2022). Insight into melatonin-mediated response and signaling in the regulation of plant defense under biotic stress. Plant Mol. Biol..

[25] Li K., Zhong C.S., Shi Q.H., Bi H.G., Gong B. (2021). Cold plasma seed treatment improves chilling resistance of tomato plants through hydrogen peroxide and abscisic acid signaling pathway. Free Radic. Biol. Med..

[26] Matern U., Kneusel R.E. (1988). Phenolic compounds in plant disease resistance. Phytoparasitica.

[27] Yahya M., Saeed N.A., Nadeem S., Hamed M., Saleem K. (2020). Effect of leaf rust disease on photosynthetic rate, chlorophyll contents and grain yield of wheat. Arch. Phytopathol. Plant Prot..

[28] Huang X., Wang H.K., Luo W.J., Xue S., Hayat F., Gao Z.H. (2021). Prediction of loquat soluble solids and titratable acid content using fruit mineral elements by artificial neural network and multiple linear regression. Sci. Hortic..

[29] Musacchi S., Serra S. (2018). Apple fruit quality: Overview on pre-harvest factors. Sci. Hortic..

[30] Rosa M., Prado C., Podazza G., Interdonato R., González J.A., Hilal M., Prado F.E. (2009). Soluble sugars: Metabolism, sensing and abiotic stress: A complex network in the life of plants. Plant Signal Behavi..

[31] Han Q., Gao H.Y., Chen H.J., Fang X.J., Wu W.J. (2017). Precooling and ozone treatments affects postharvest quality of black mulberry (*Morus nigra*) fruits. Food Chem..

[32] Fenech M., Amaya I., Valpuesta V., Botella M.A. (2019). Vitamin C content in fruits: Biosynthesis and regulation. Front. Plant Sci..

[33] Wang Z.J., Li Y.H., Zhang H.Y., Yan X.X., Cui H. (2022). Methyl jasmonate treatment, aphid resistance assay, and transcriptomic analysis revealed different herbivore defensive roles between tobacco glandular and non-glandular trichomes. Plant Cell Rep..

[34] Shen Y.T., Ma Y.L., Li D.Y., Kang M.M., Pei Y., Zhang R., Tao W.Y., Huang S.X., Song W.J., Li Y.C. (2023). Biological and genomic analysis of a symbiotic nitrogen fixation defective mutant in *Medicago truncatula*. Front. Plant Sci..

[35] Kusajima M. (2019). Studies on the mechanism of agricultural chemicals focused on plant hormone signals. J. Pestic. Sci..

[36] Alazem M., Lin N. (2017). Antiviral roles of abscisic acid in plants. Front. Plant Sci..

[37] Lin Z., Li Y., Wang Y., Liu X., Ma L., Zhang Z., Mu C., Zhang Y., Peng L., Xie S. (2021). Initiation and amplification of SnRK2 activation in abscisic acid signaling. Nat. Commun..

[38] García-Andrade J., González B., Gonzalez-Guzman M., Rodriguez P.L., Vera P. (2020). The role of ABA in plant immunity is mediated through the PYR1 receptor. Int. J. Mol. Sci..

[39] Ma Y., Szostkiewicz I., Korte A., Moes D., Yang Y., Christmann A., Grill E. (2009). Regulators of PP2C phosphatase activity function as abscisic acid sensors. Science.

[40] Park S., Fung P., Nishimura N., Jensen D.R., Fujii H., Zhao Y., Lumba S., Santiago J., Rodrigues A., Chow T.F. (2009). Abscisic acid inhibits type 2C protein phosphatases via the PYR/PYL family of START proteins. Science.

[41] Zhang W., Guo S.X., Wang Y., Wu Y.K., Yu L.J., Wu J. (2023). Trifluoromethylpyridine piperazine derivatives: Synthesis and anti-plant virus activity. Pest Manag. Sci..

[42] Montasser M.S., Tousignant M.E., Kaper J.M. (1998). Viral satellite RNAs for the prevention of cucumber mosaic virus (CMV) disease in field-grown pepper and melon plants. Plant Dis..

[43] Haroon E.T., Zou X.B., Li Z.H., Zhu Y.D. (2015). Comprehensive evaluation of antioxidant properties and volatile compounds of sudanese honeys. J. Food Biochem..

[44] Vidović M., Morina F., Milić S., Albert A., Zechmann B., Tosti T., Winkler J.B., Jovanović S.V. (2015). Carbon allocation from source to sink leaf tissue in relation to flavonoid biosynthesis in variegated Pelargonium zonale under UV-B radiation and high PAR intensity. Plant Physiol. Biochem..

[45] Farhadi H., Babaei K., Farsaraei S., Moghaddam M., Pirbalouti A.G. (2020). Changes in essential oil compositions, total phenol, flavonoids and antioxidant capacity of *Achillea millefolium* at different growth stages. Ind. Crop. Prod..

[46] Zhao J., Zhou J.J., Wang Y.Y., Gu J.W., Xie X.Z. (2013). Positive regulation of phytochrome B on chlorophyll biosynthesis and chloroplast development in rice. Rice Sci..

[47] Maryam A., Anwar R., Malik A.U., Khan S.A. (2021). Influence of macro-perforated polyethylene terephthalate and low-density polyethylene packaging films on quality and storability of strawberries. J. Food Process. Pres..

[48] Johnson J.B., Mani J.S., Hoyos B.E., Naiker M. (2023). Phenolic profiles, phytochemical composition and vitamin C content of selected horticultural produce from Central Queensland. J. Food Meas. Charact..

[49] Qu X.L., Zhu K.X., Li Z.X., Zhang D.F., Hou L.J. (2021). The alteration of M6A-tagged transcript profiles in the retina of rats after traumatic optic neuropathy. Front. Genet..

[50] Moon S., Zhao Y.T. (2021). Spatial, temporal and cell-type-specific expression profiles of genes encoding heparan sulfate biosynthesis enzymes and proteoglycan core proteins. Glycobiology.

[51] Zhang H.P., Zou J., Yin Y., Zhang B., Hu Y.L., Wang J.J., Mu H.J. (2019). Bioinformatic analysis identifies potentially key differentially expressed genes in oncogenesis and progression of clear cell renal cell carcinoma. PeerJ.

[52] Yu L., Wang W.L., Zeng S., Chen Z., Yang A.M., Shi J. (2018). Label-free quantitative proteomics analysis of cytosinpeptidemycin responses in southern rice blackstreaked dwarf virus-infected rice. Pestic. Biochem. Phys..

