# Supplementary material for: Novel 4-Chromanone-Derived Compounds as Plant Immunity Inducers against CMV Disease in Passiflora spp. (Passion Fruit)
Source: Molecules. 2024 Feb 28;29(5):1045. doi: 10.3390/molecules29051045 (PMC10934444; doi:10.3390/molecules29051045)
Supplement: Supplementary file 1 [file molecules-29-01045-s001.zip › Supplementary material.pdf]

Supplementary material

Novel 4-Chromanone-Derived Compounds as Plant Immunity Inducers against CMV Disease in *Passiflora* spp. (Passion Fruit)

Tianli Wu [1,†], Lu Yu [1,*,†], Lingling Xiao [1,2], Tao Wang [2,*], Pei Li [1,3,*] and Bo Mu [4]

[1] School of Liquor and Food Engineering, Guizhou University, Guiyang 550025, China; w1612281228@163.com (T.W.); an1378386891@163.com (L.X.)
[2] Guizhou Light Industry Technical College, Guiyang 550032, China
[3] Qiandongnan Engineering and Technology Research Center for Comprehensive Utilization of National Medicine, Kaili University, Kaili 556011, China
[4] Guizhou Academy of Testing and Analysis, Guiyang 550000, China; 18143522730@163.com
* Correspondence: lyu1@gzu.edu.cn (L.Y.); wangtaotougao@126.com (T.W.); lipei@kluniv.edu.cn (P.L.)
† These authors contributed equally to this work.

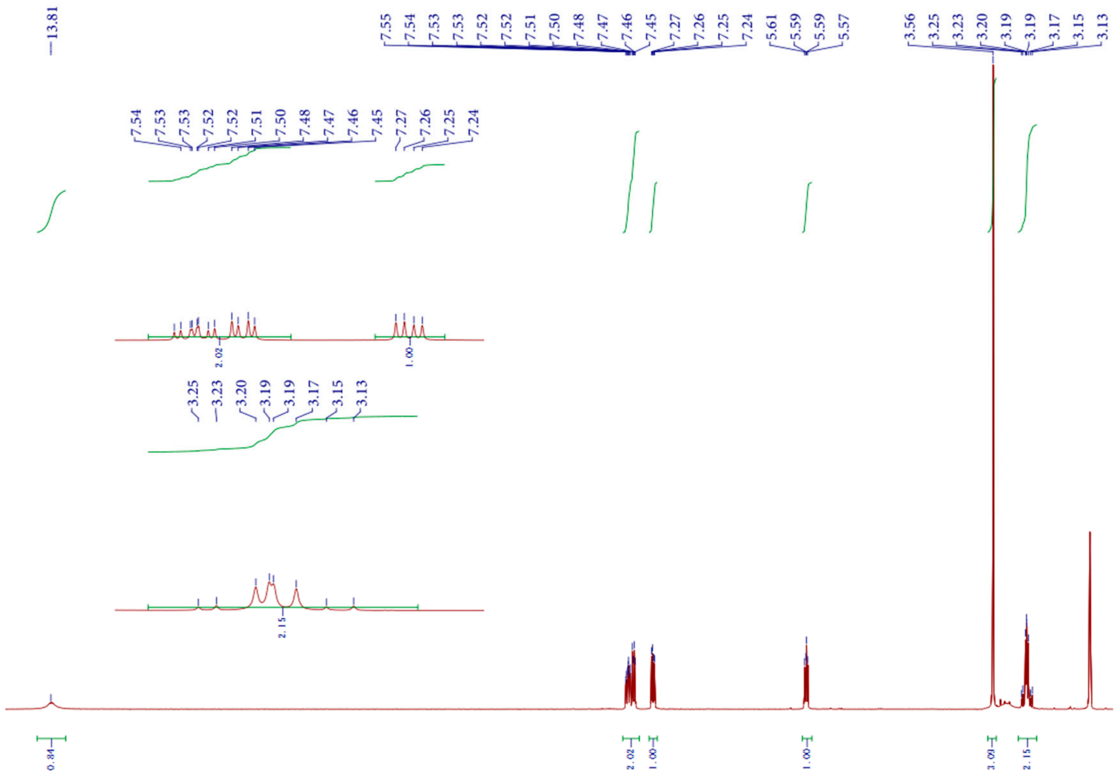

^{1}H NMR of compound 7a

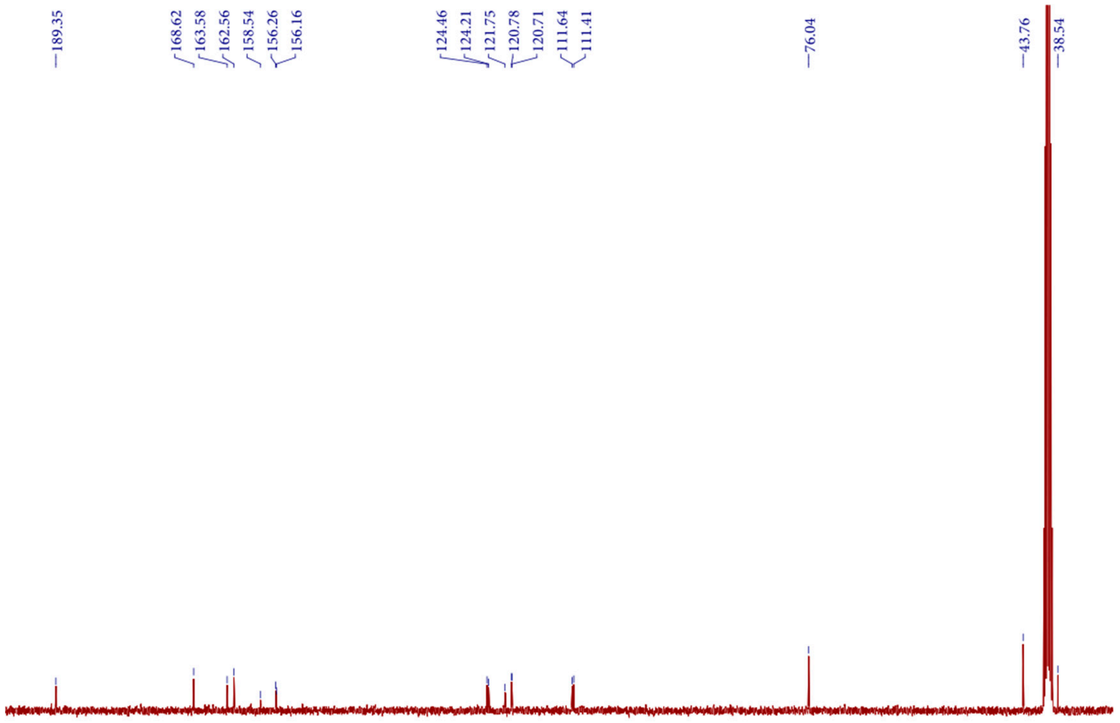

^{13}C NMR of compound 7a

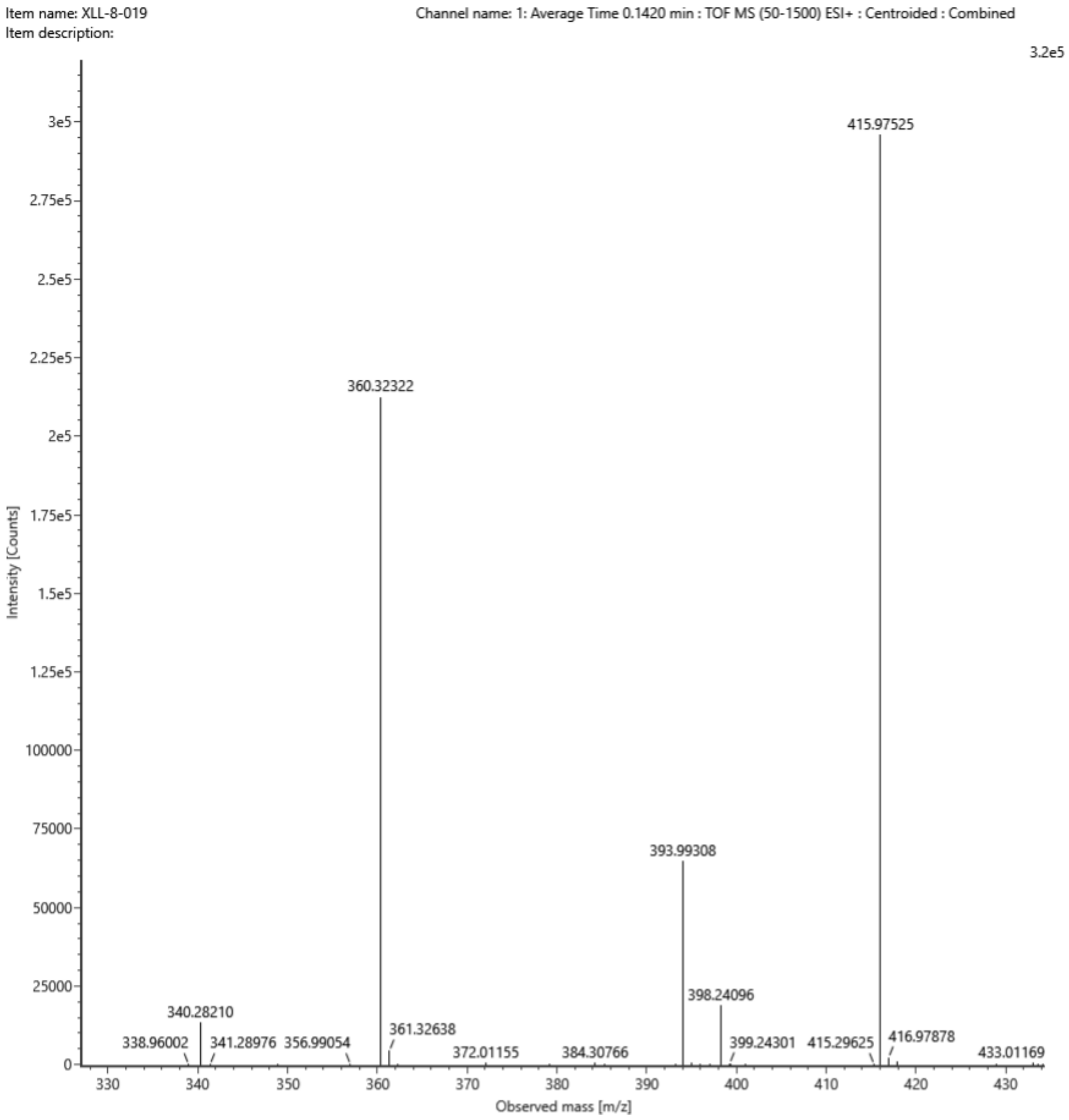

HRMS of compound 7a

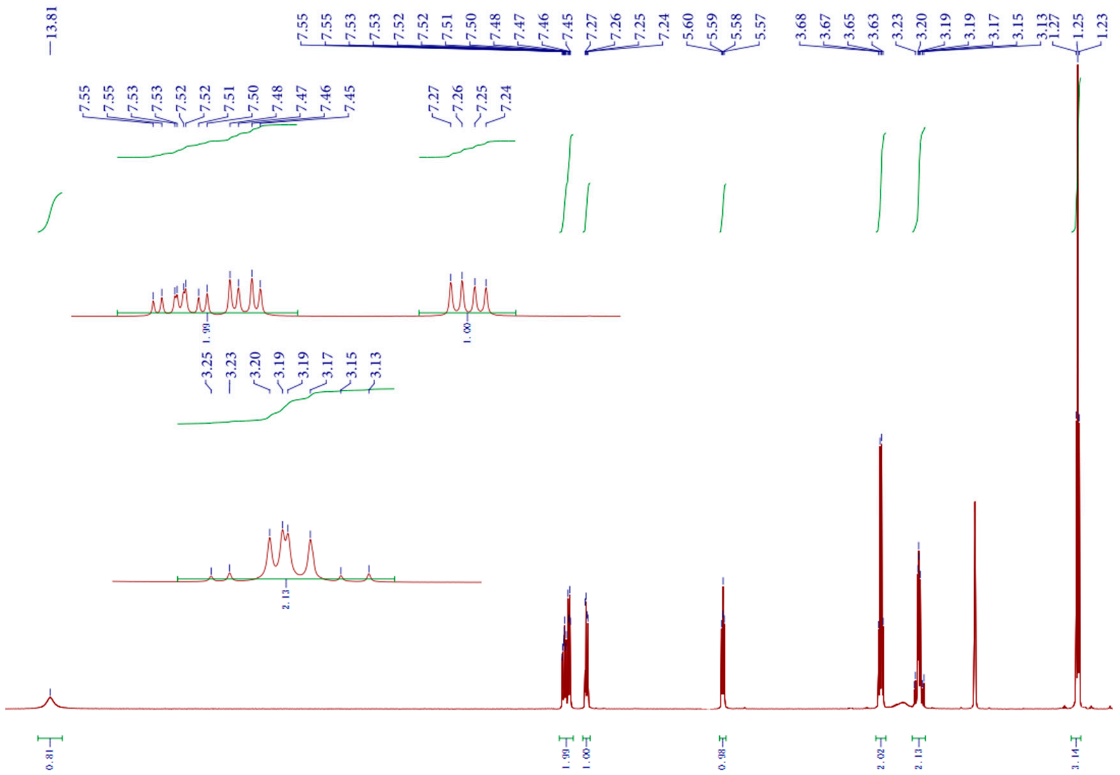

^1H NMR of compound 7b

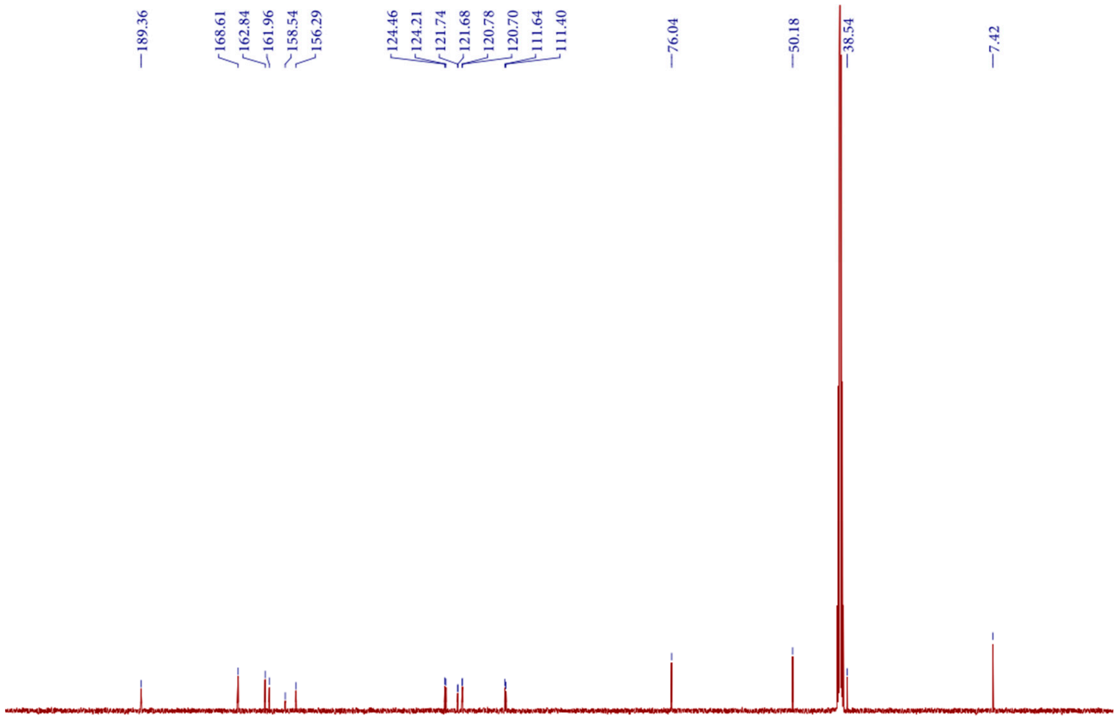

^{13}C NMR of compound 7b

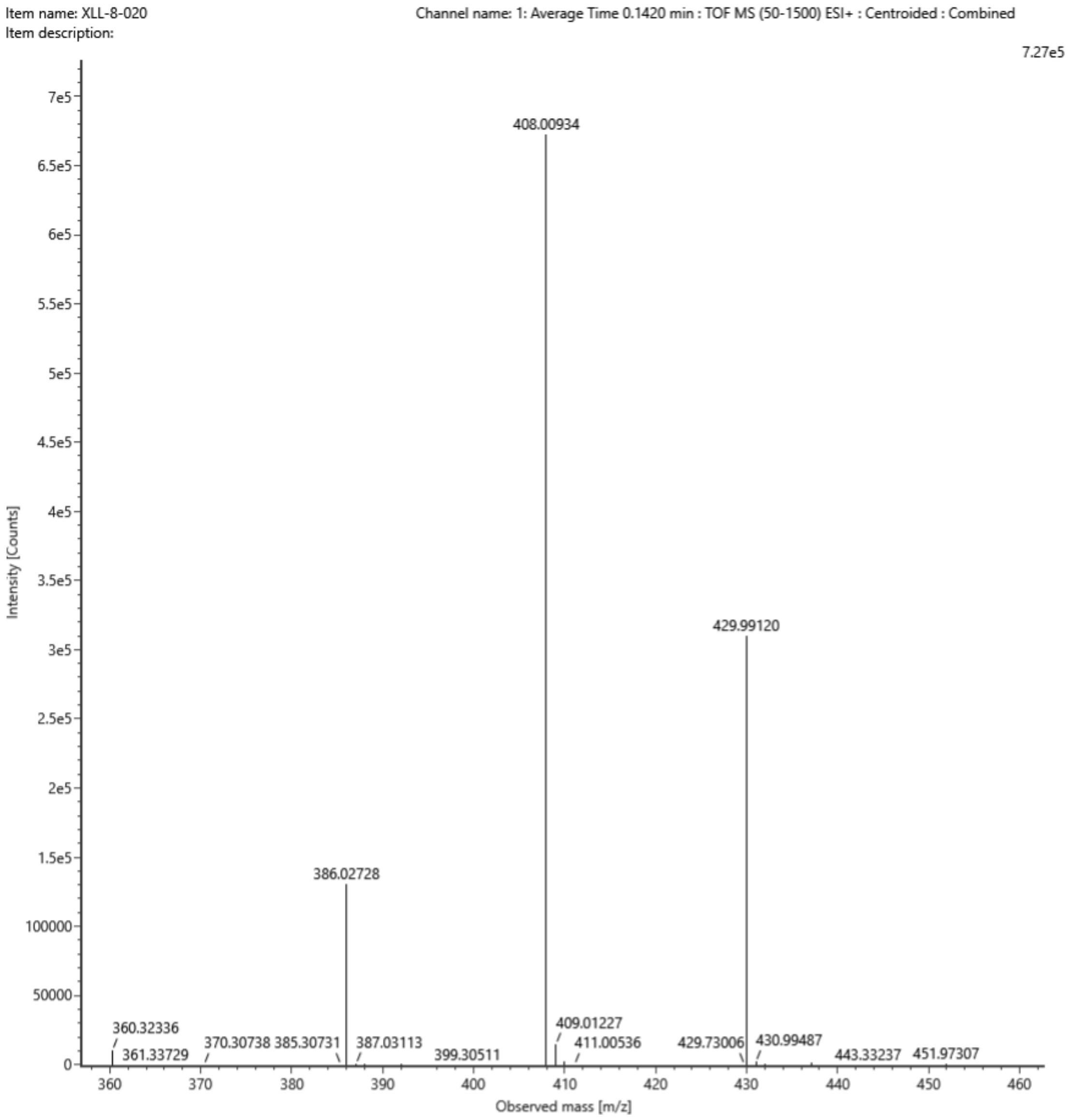

HRMS of compound 7b

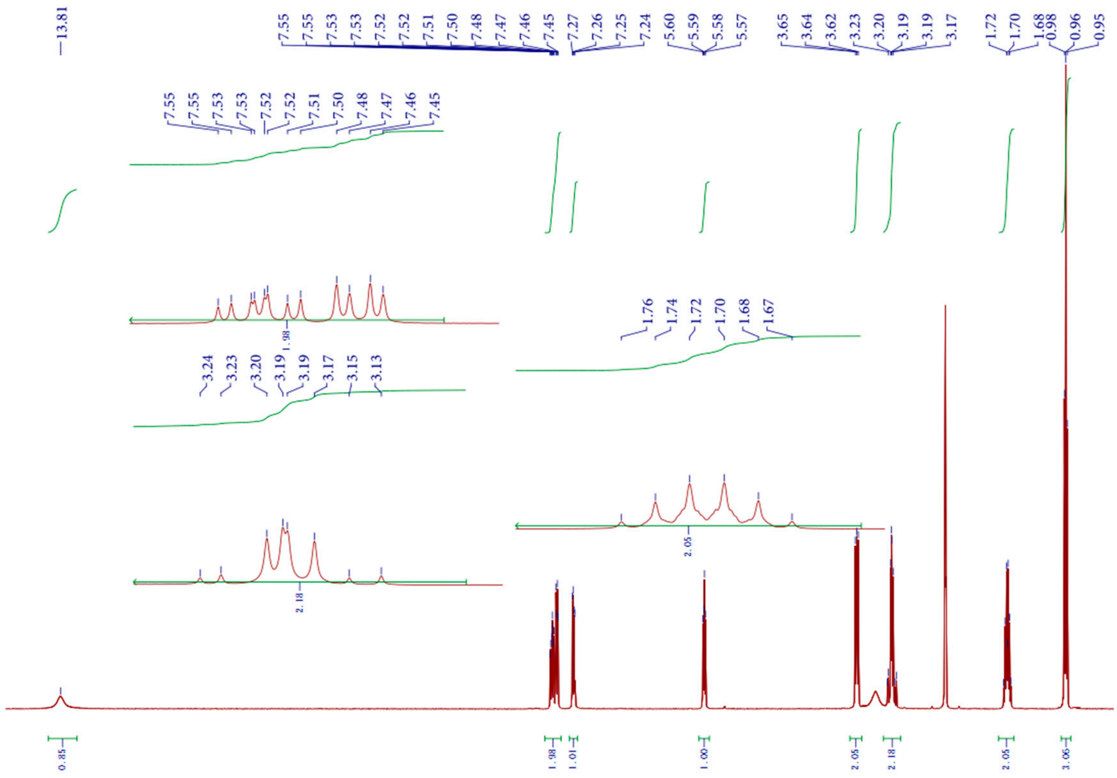

^{1}H NMR of compound 7c

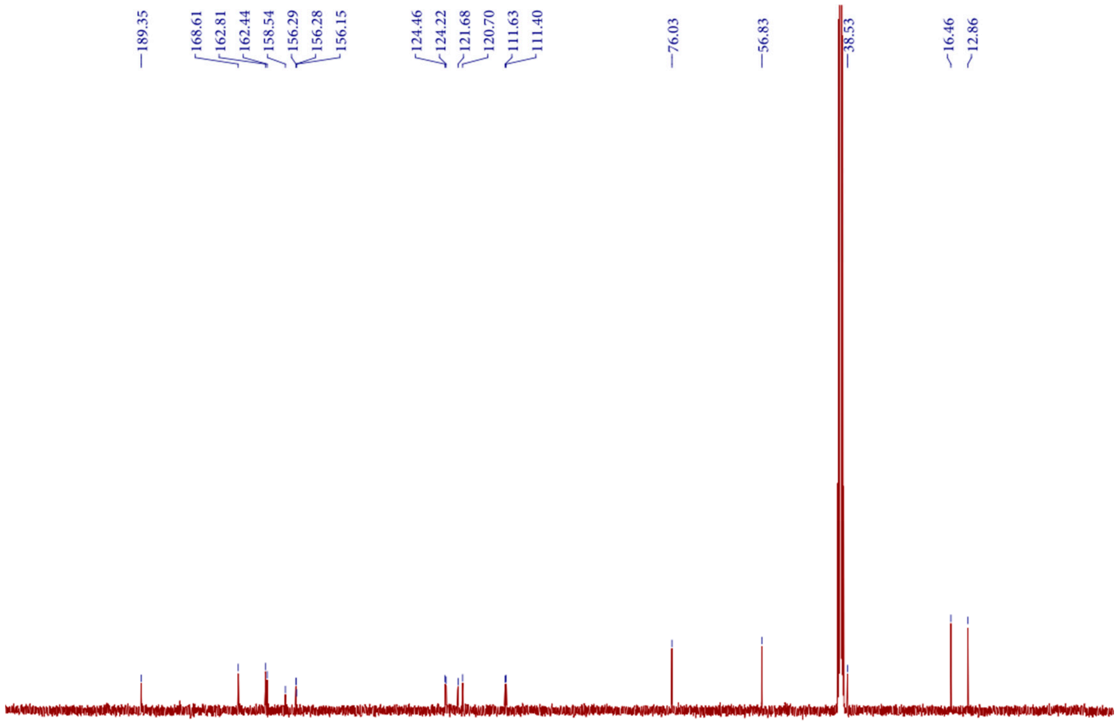

^{13}C NMR of compound 7c

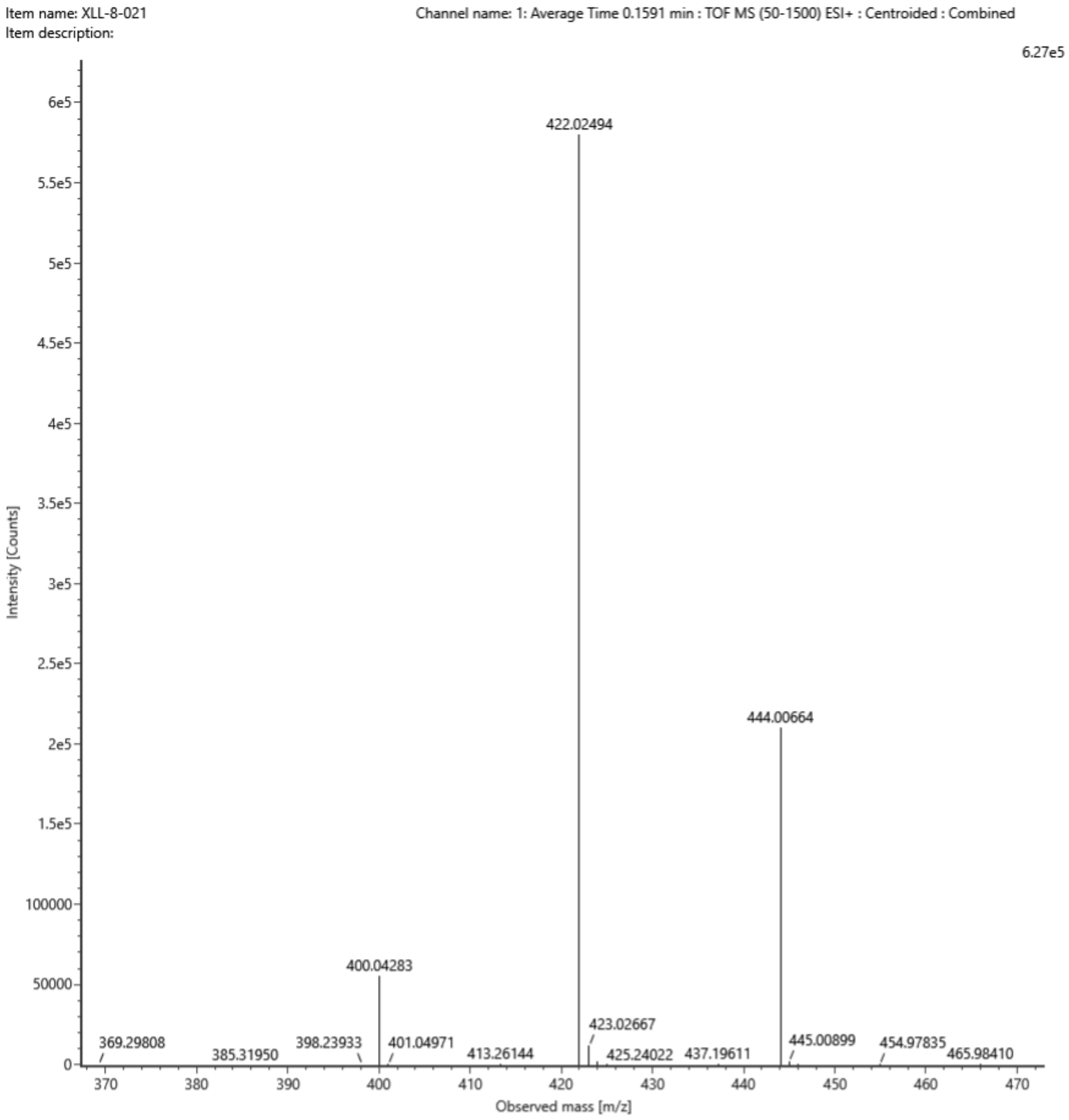

HRMS of compound 7c

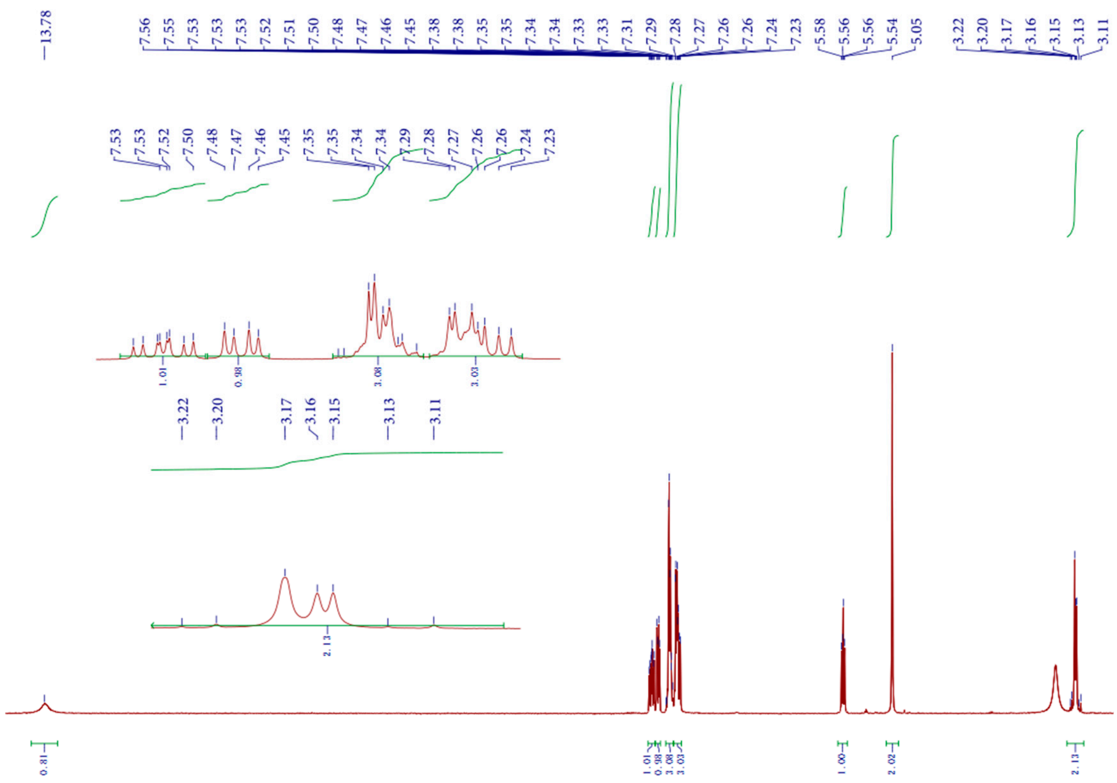

^{1}H NMR of compound 7d

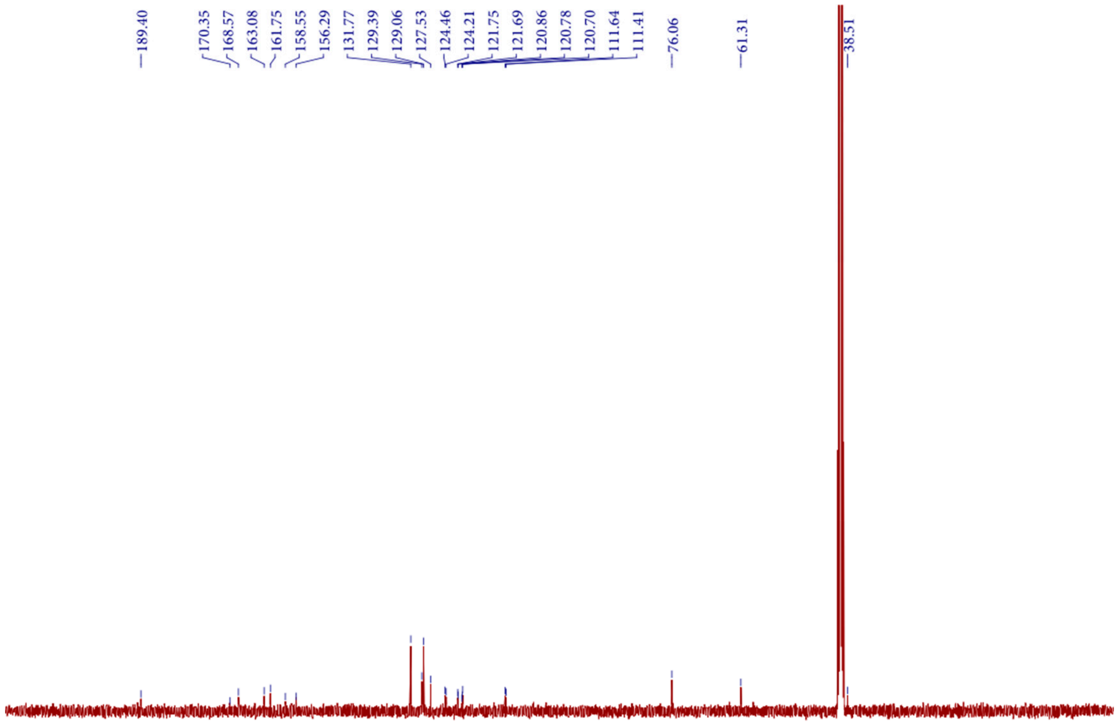

^{13}C NMR of compound 7d

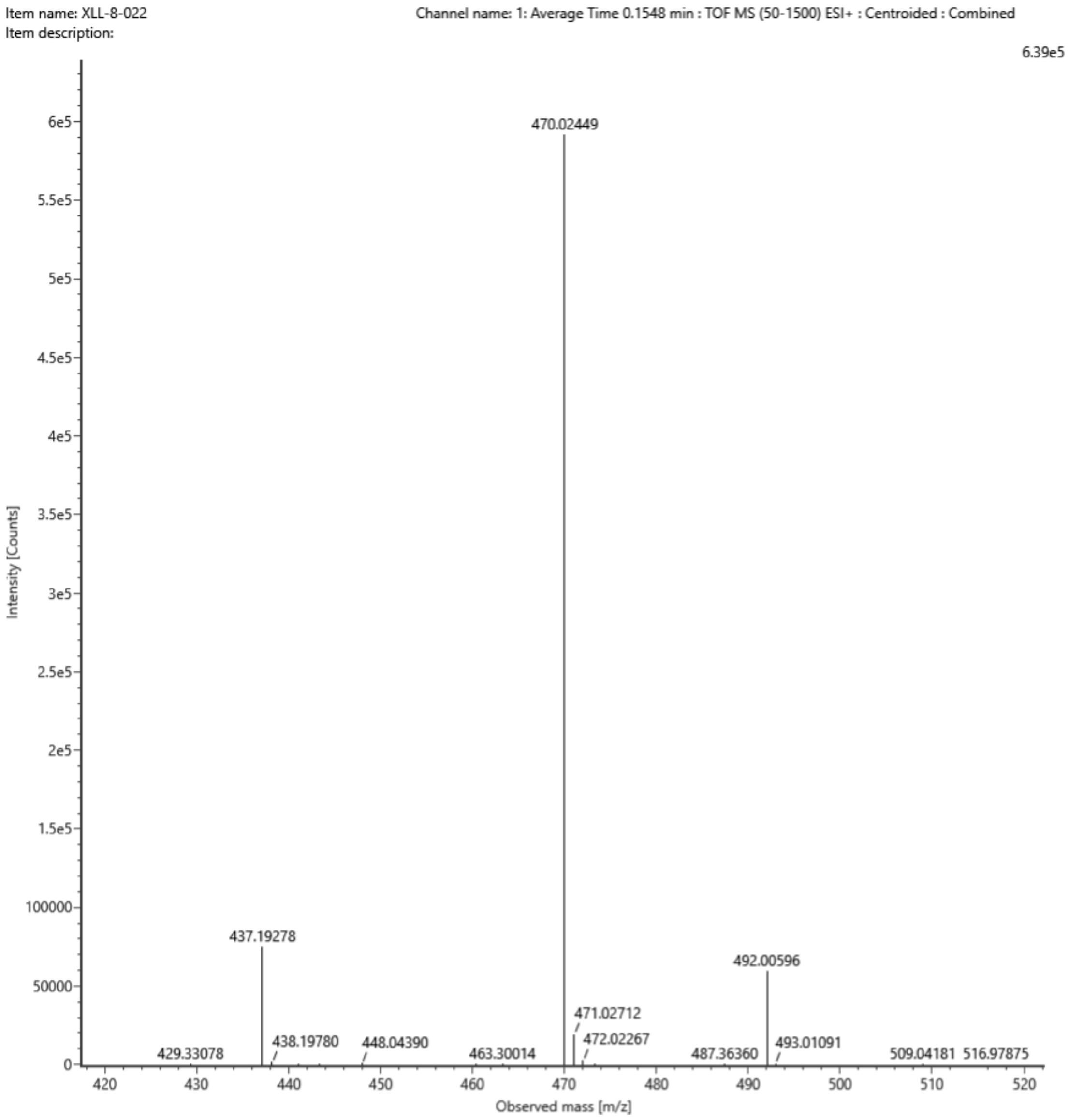

HRMS of compound 7d

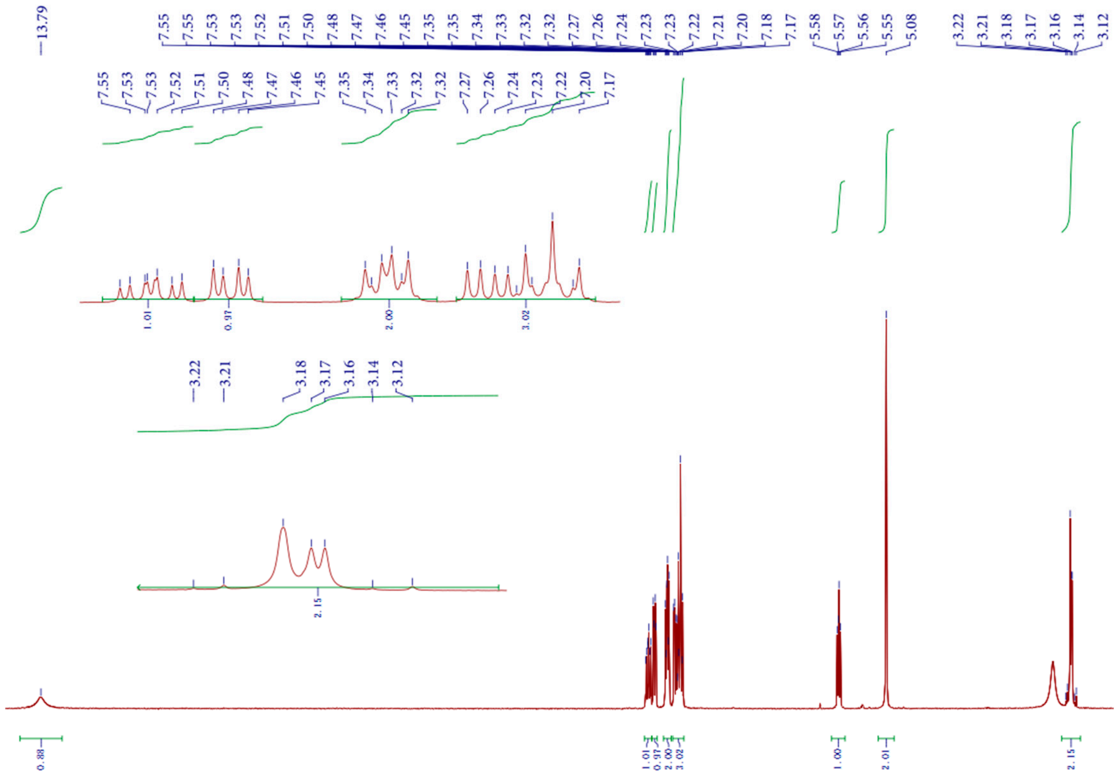

^{1}H NMR of compound 7e

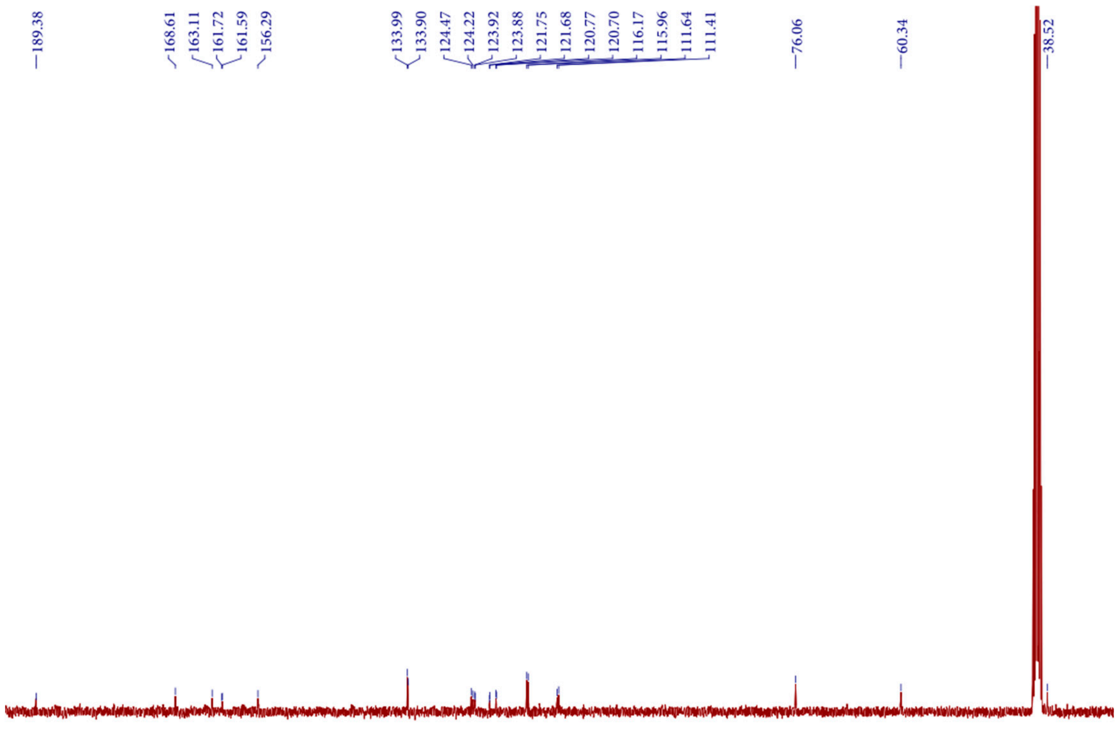

^{13}C NMR of compound 7e

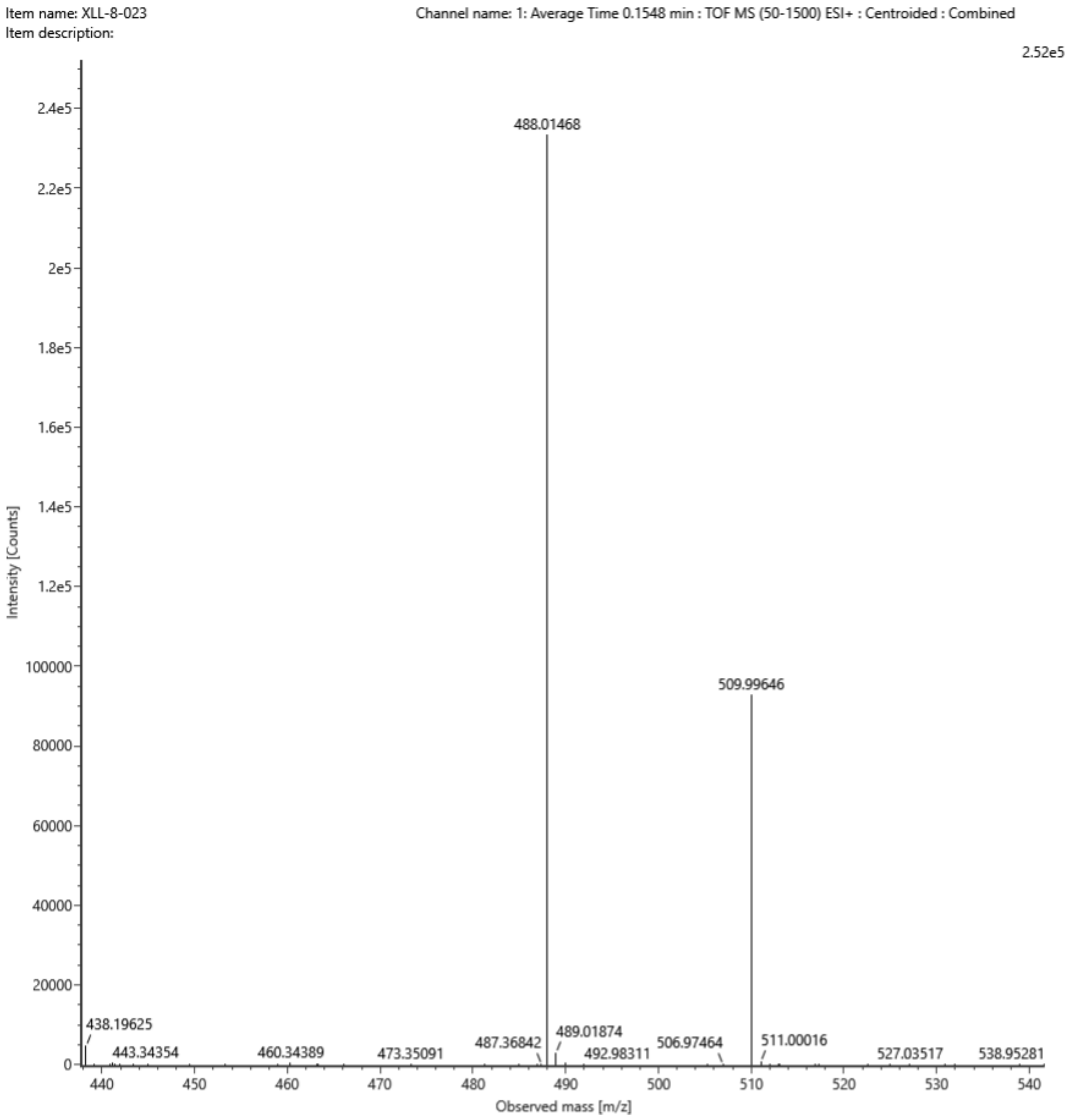

HRMS of compound 7e

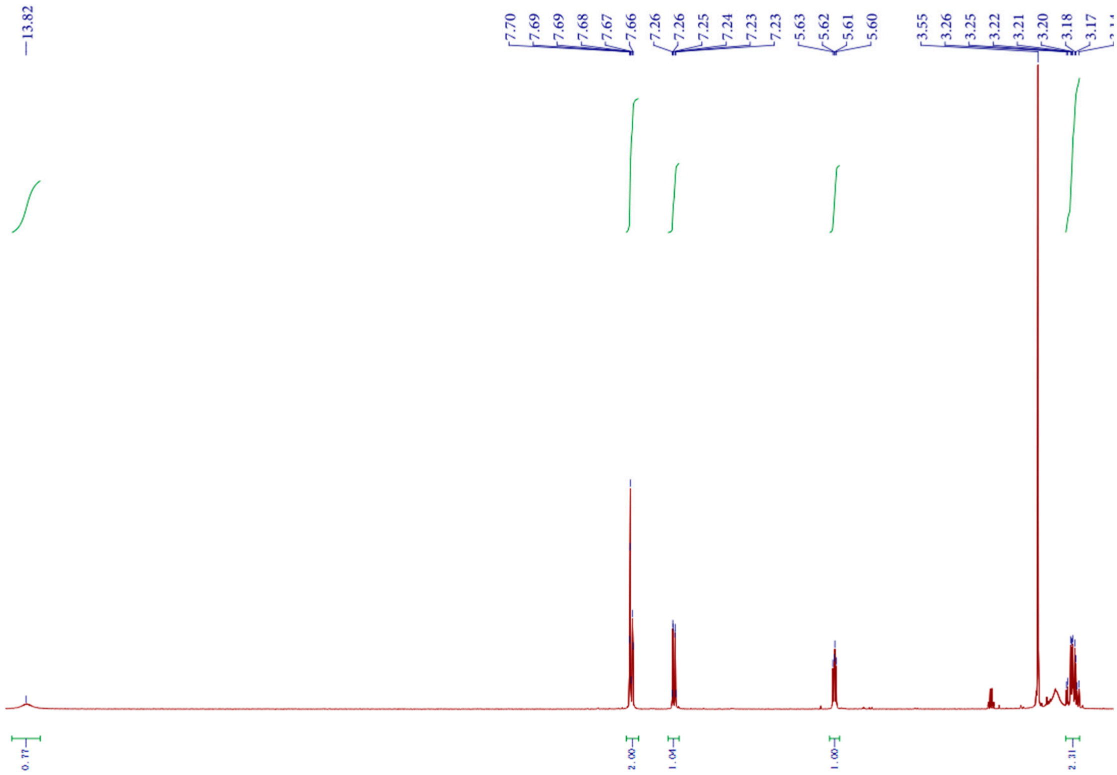

^{1}H NMR of compound 7f

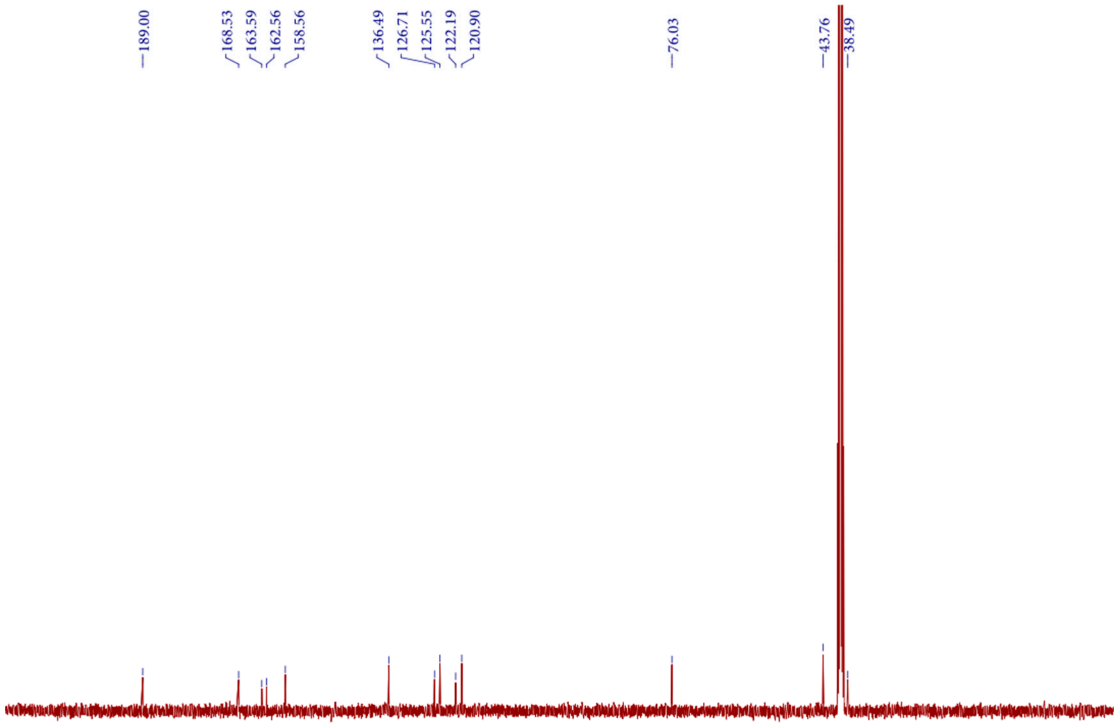

^{13}C NMR of compound 7f

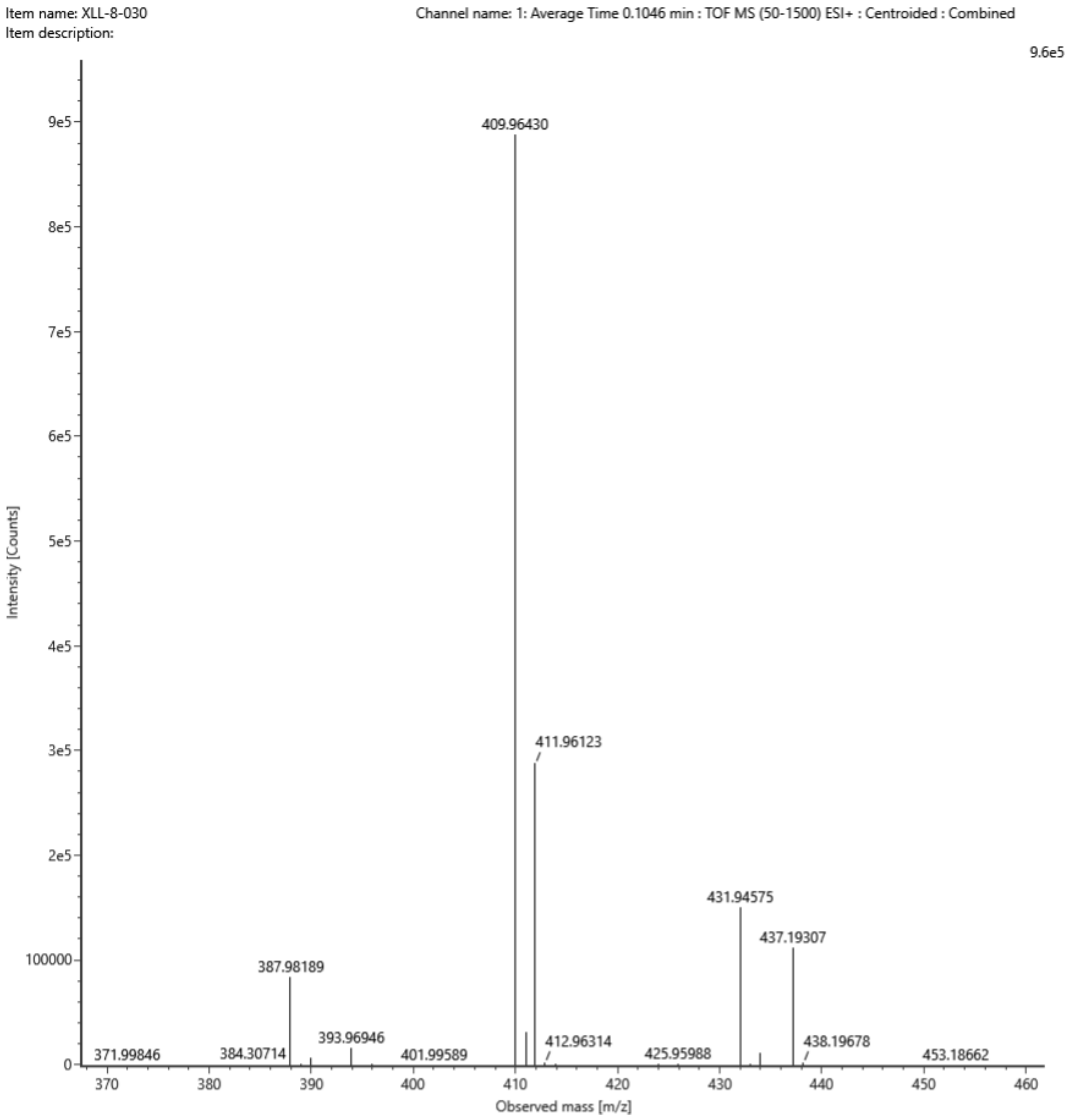

HRMS of compound 7f

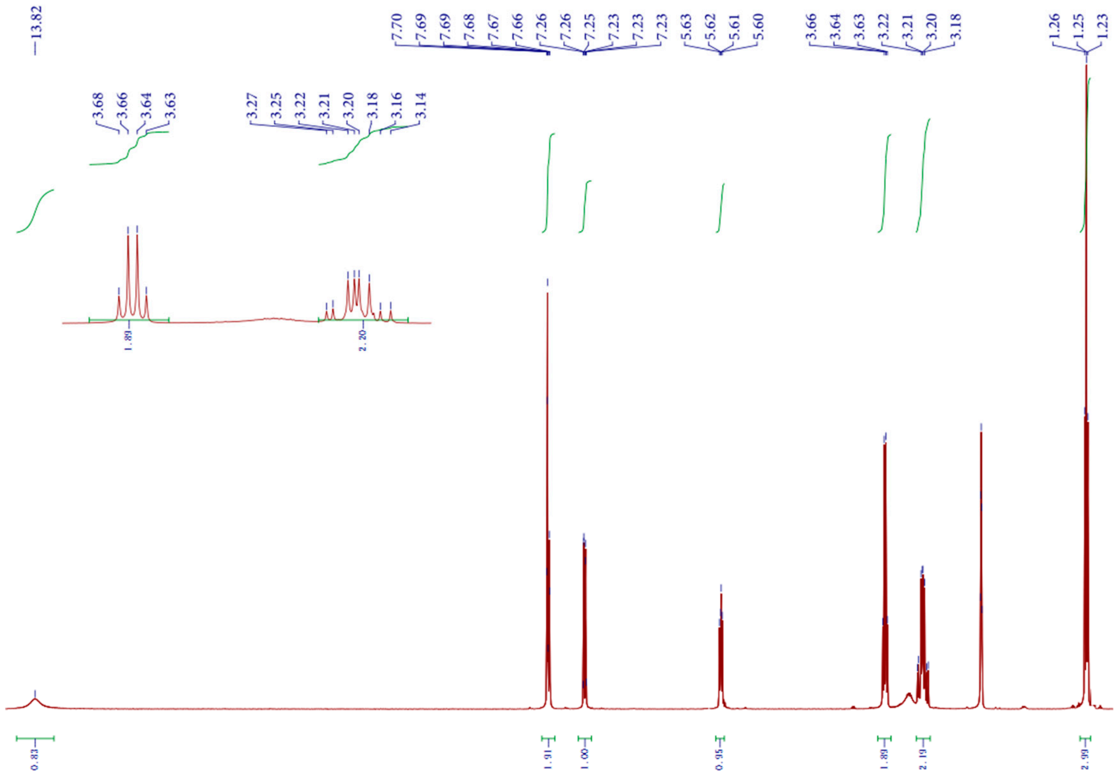

^{1}H NMR of compound 7g

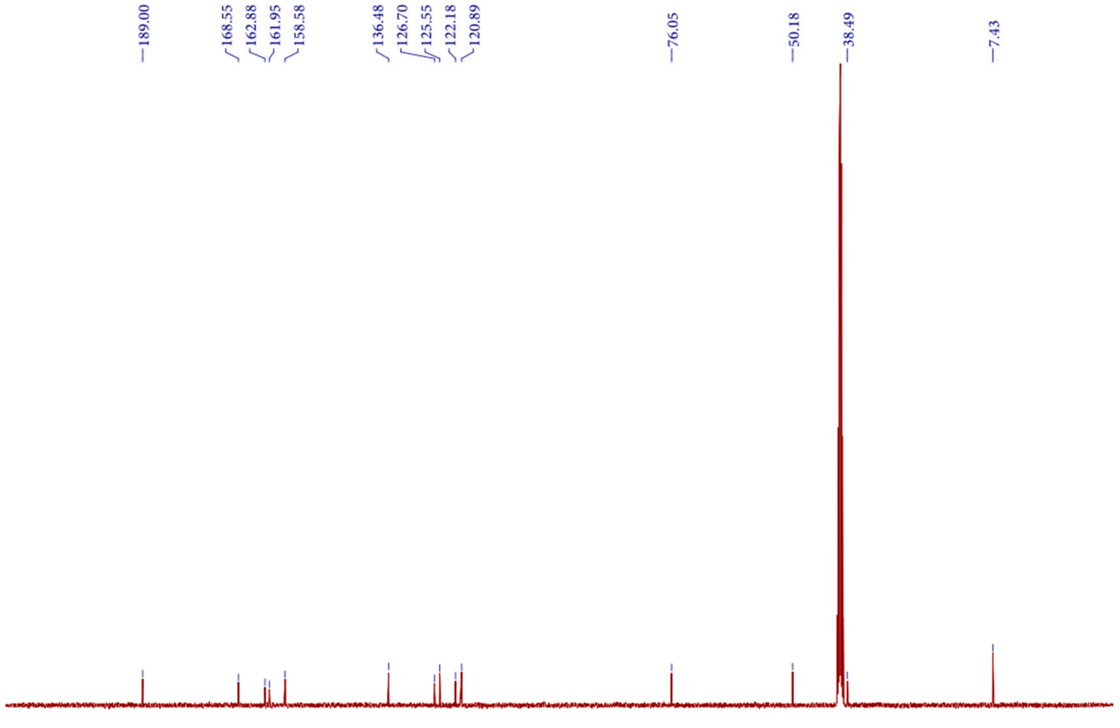

^{13}C NMR of compound 7g

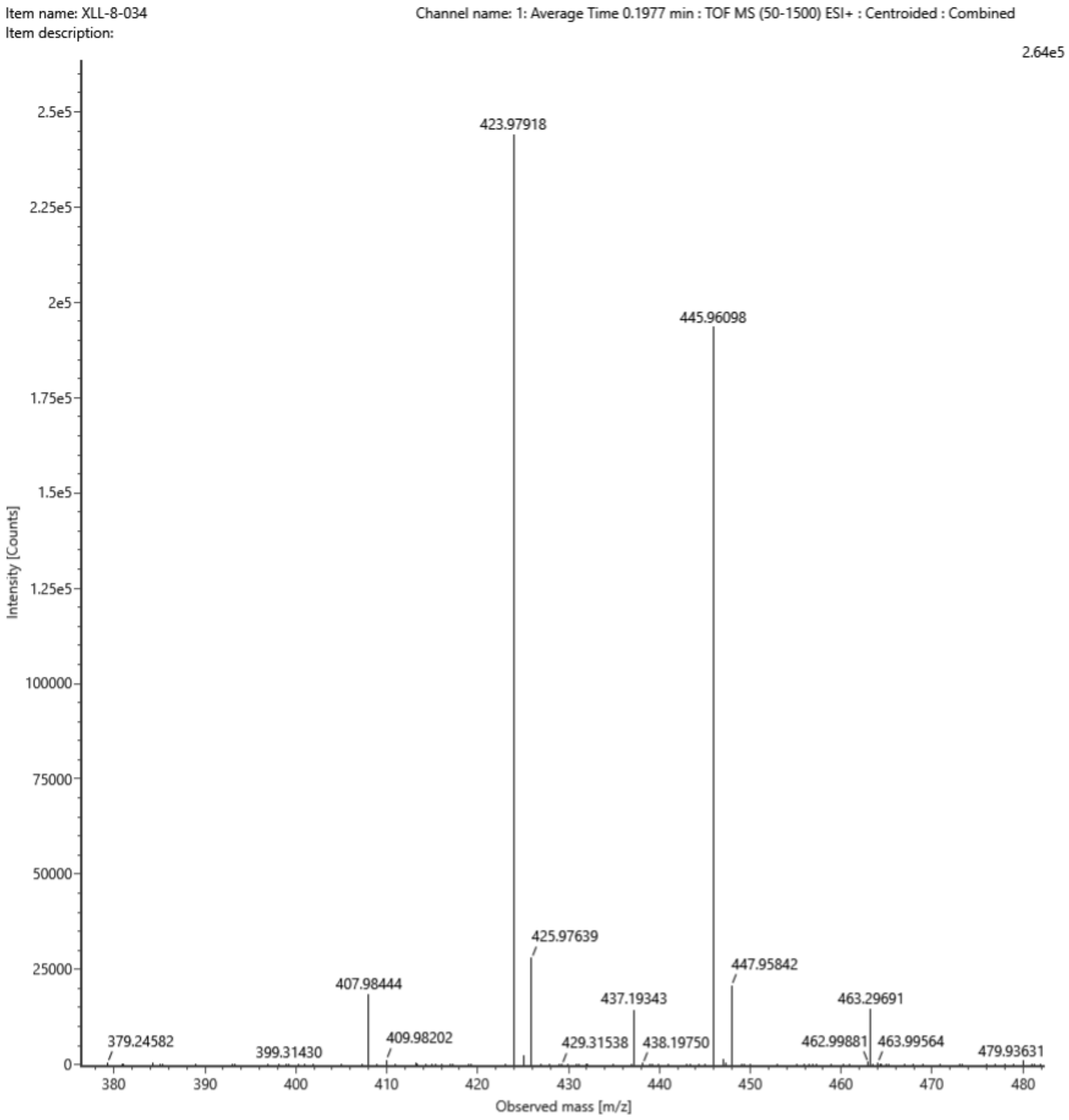

HRMS of compound 7g

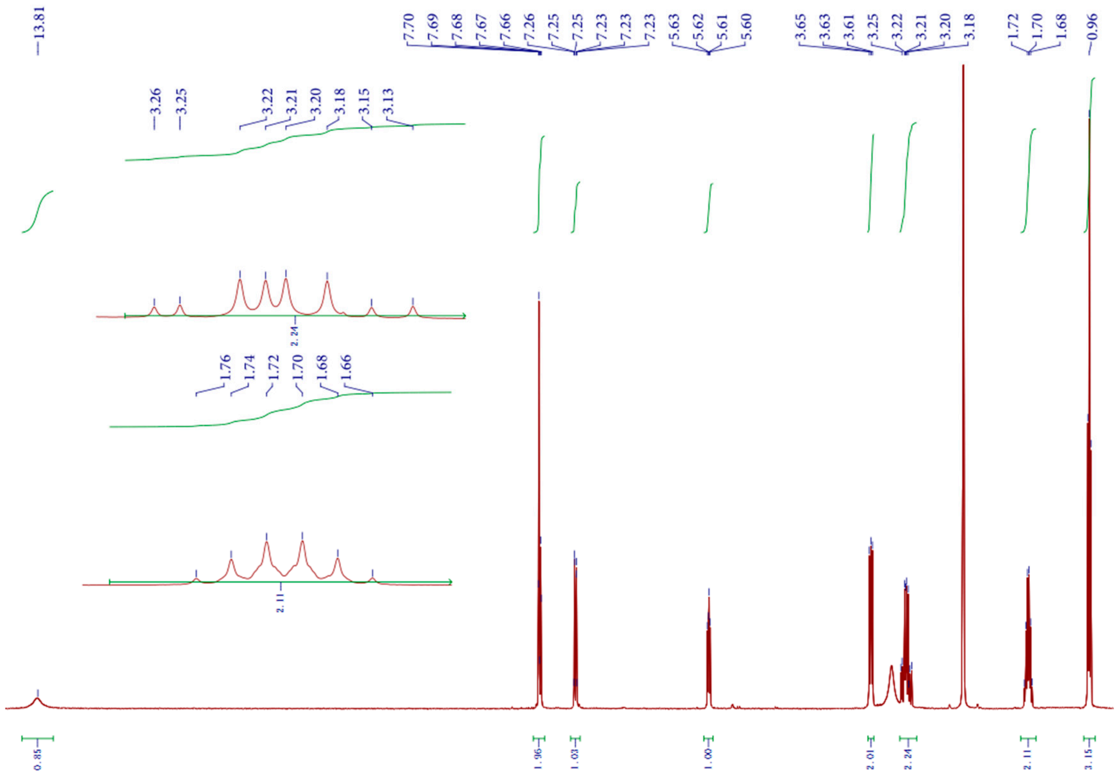

^{1}H NMR of compound 7h

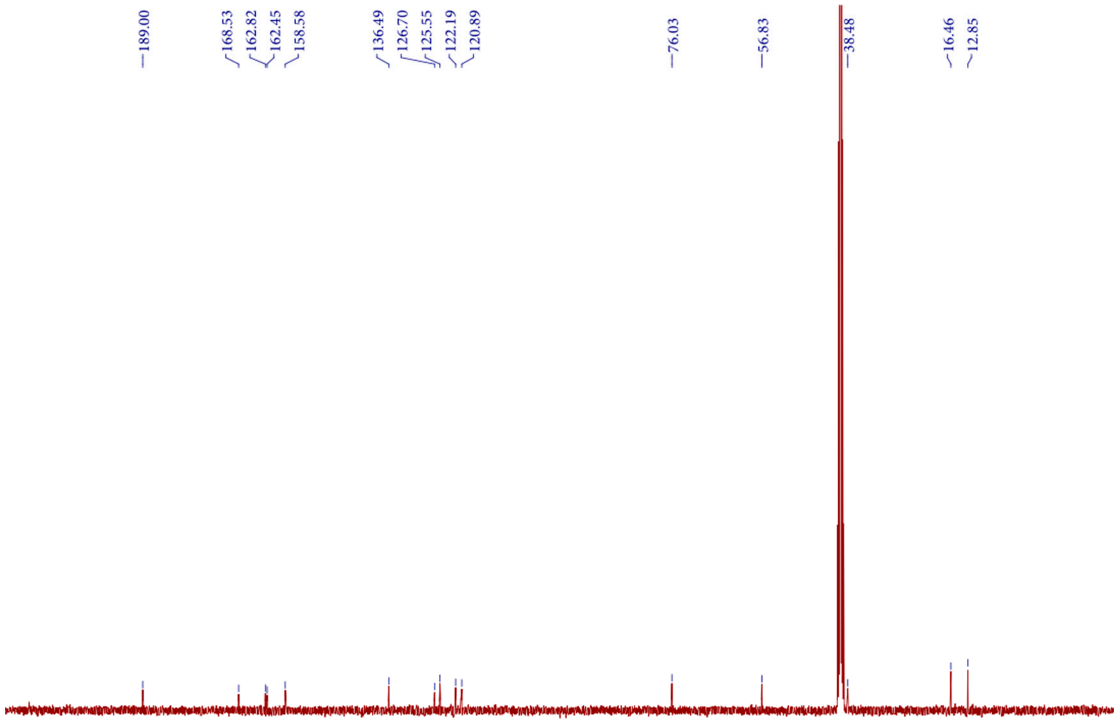

^{13}C NMR of compound 7h

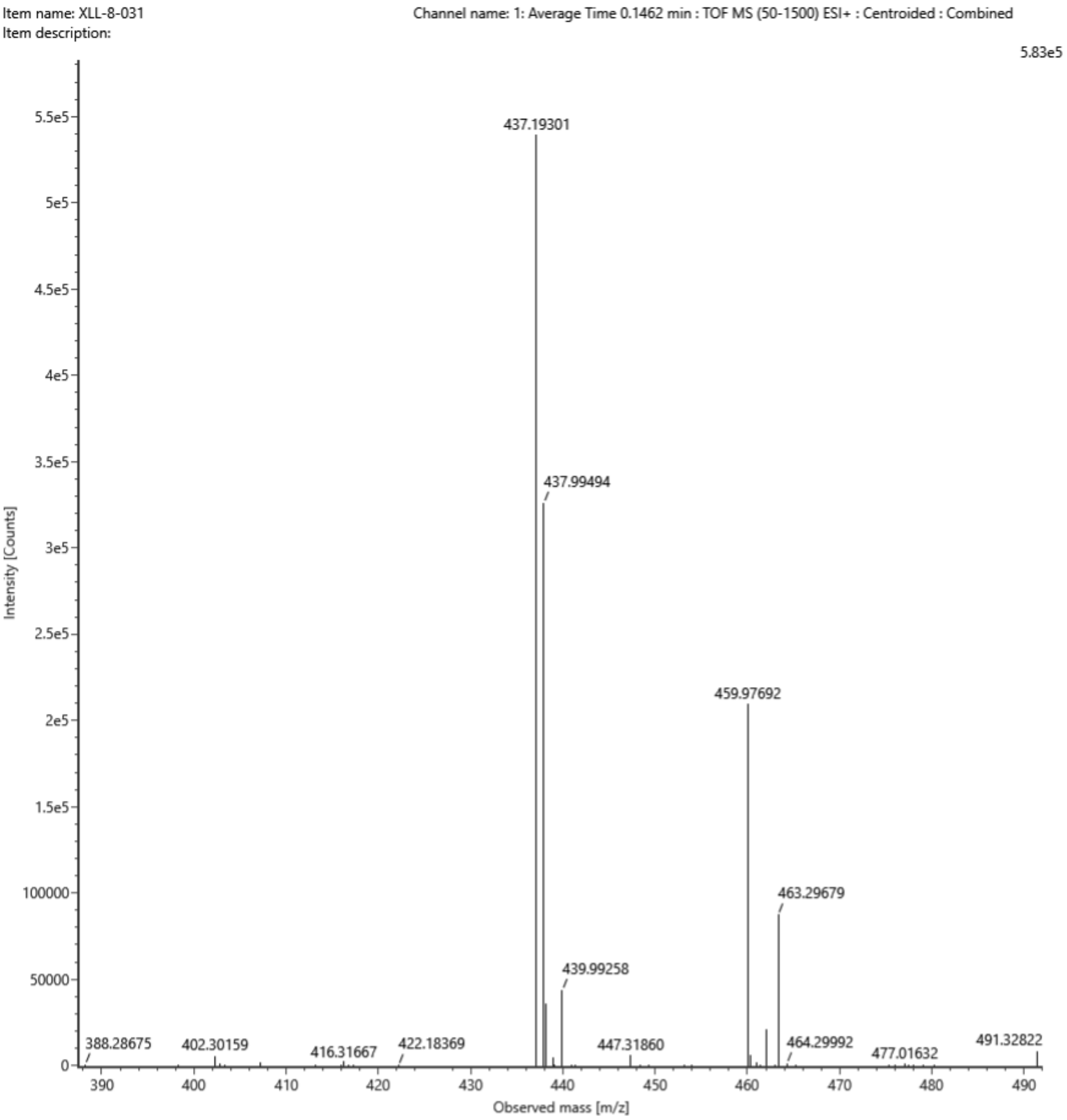

HRMS of compound 7h

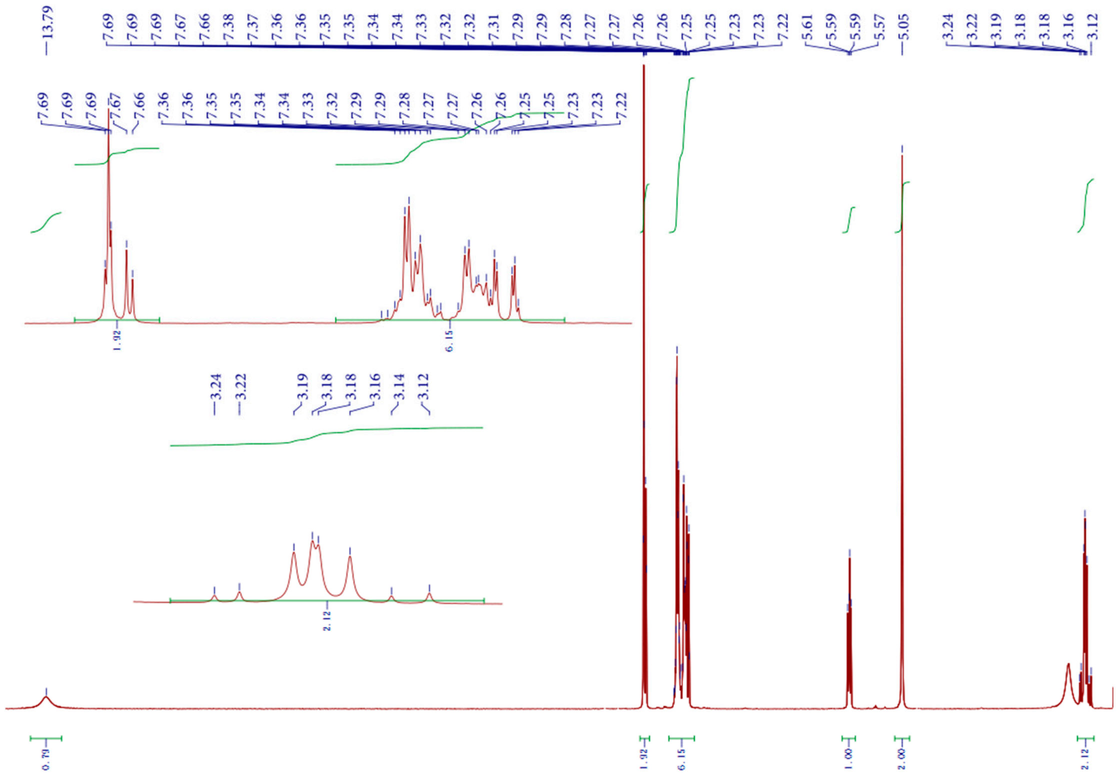

^{1}H NMR of compound 7i

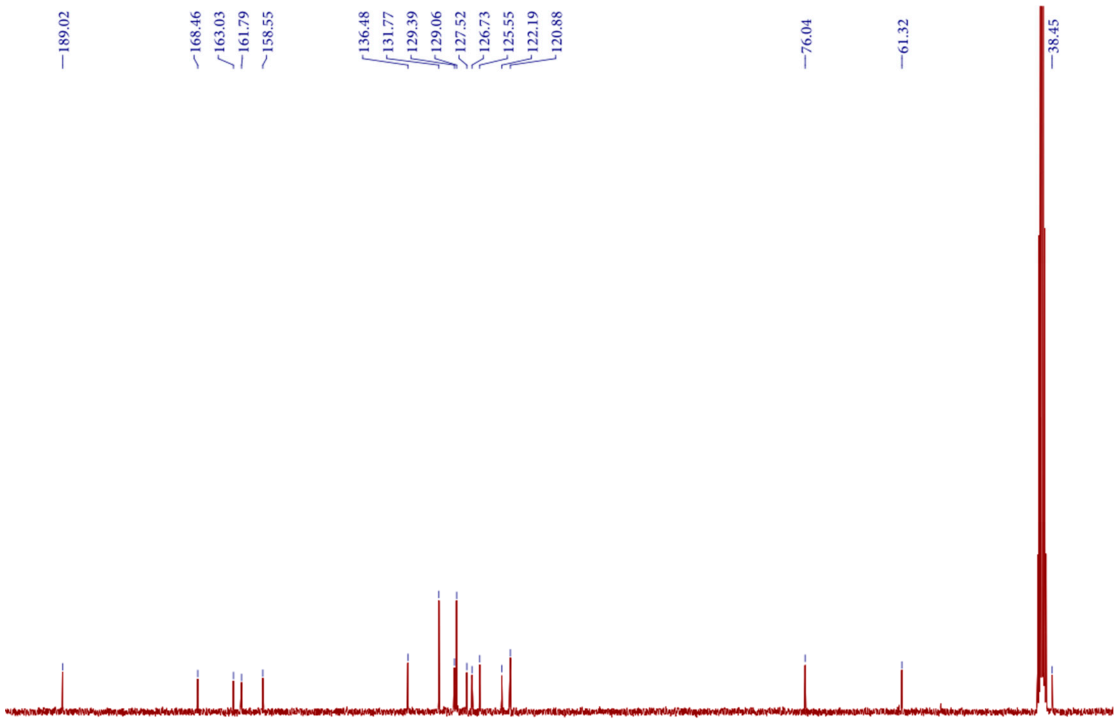

^{13}C NMR of compound 7i

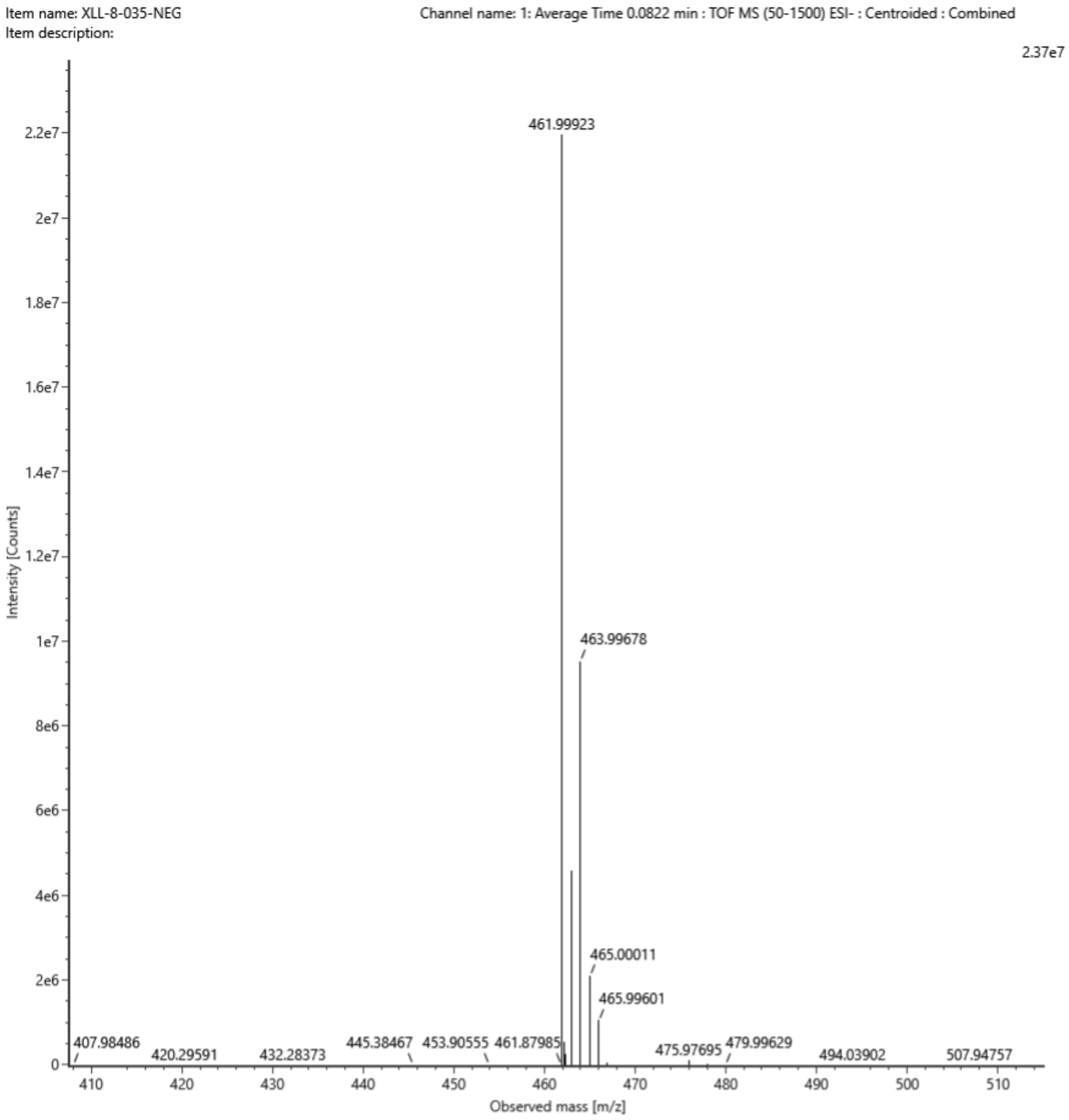

HRMS of compound 7i

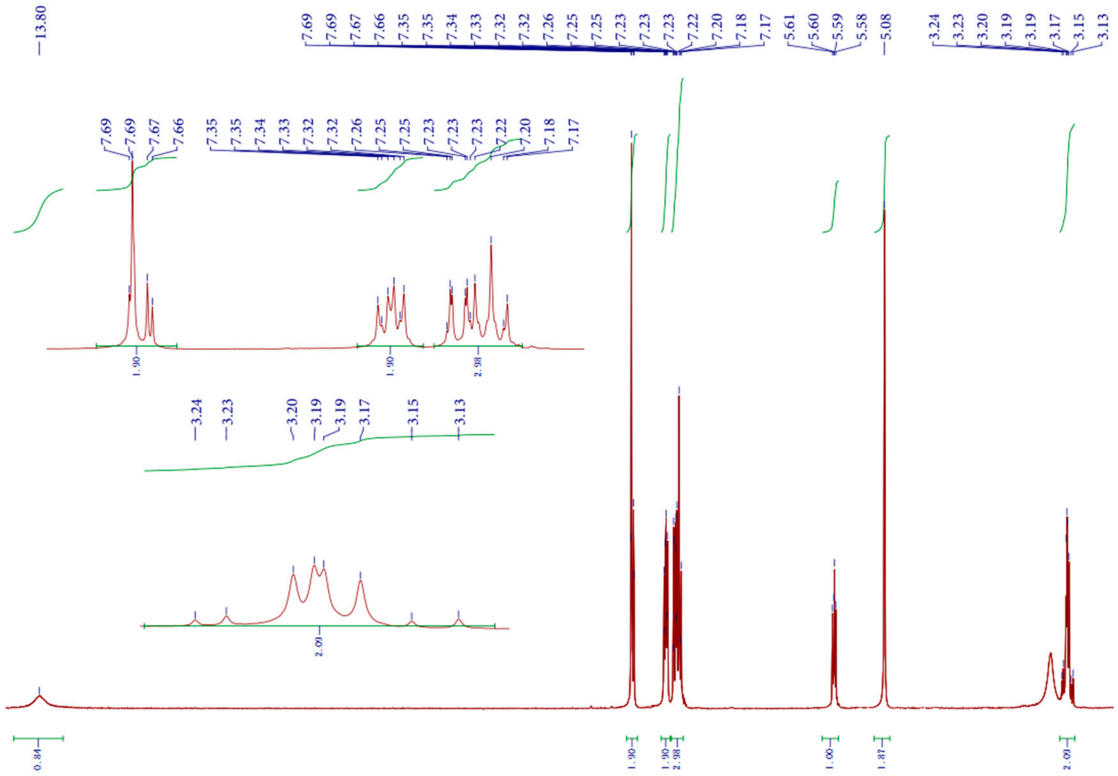

^{1}H NMR of compound 7j

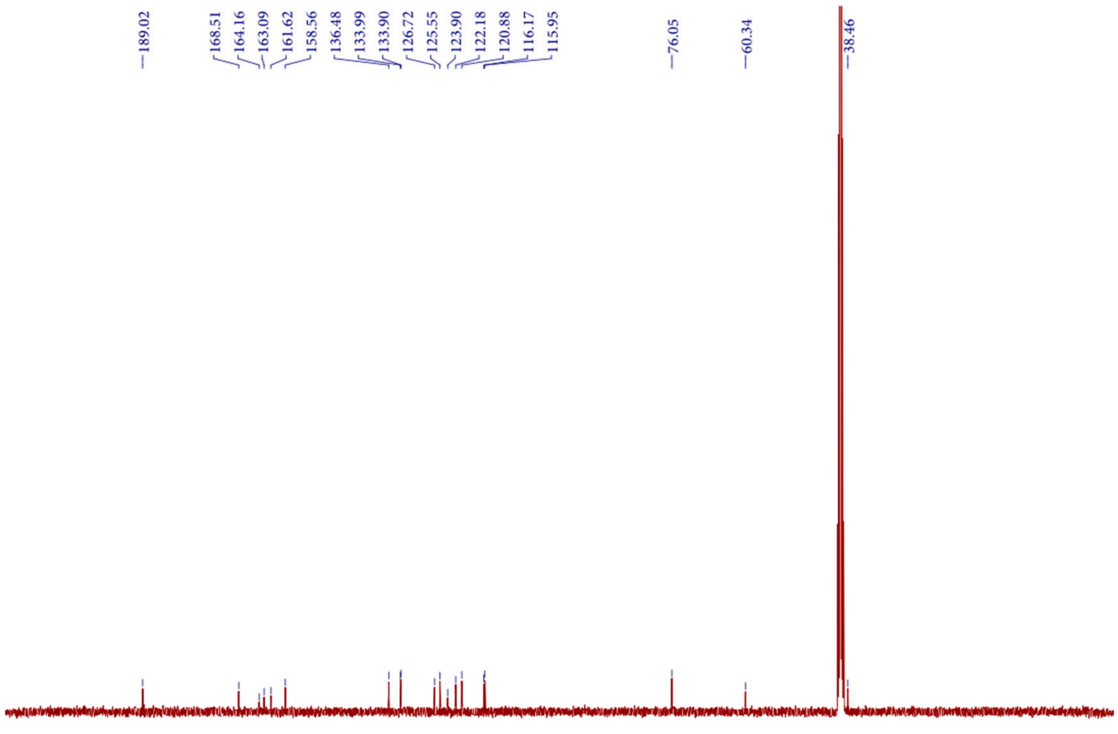

^{13}C NMR of compound 7j

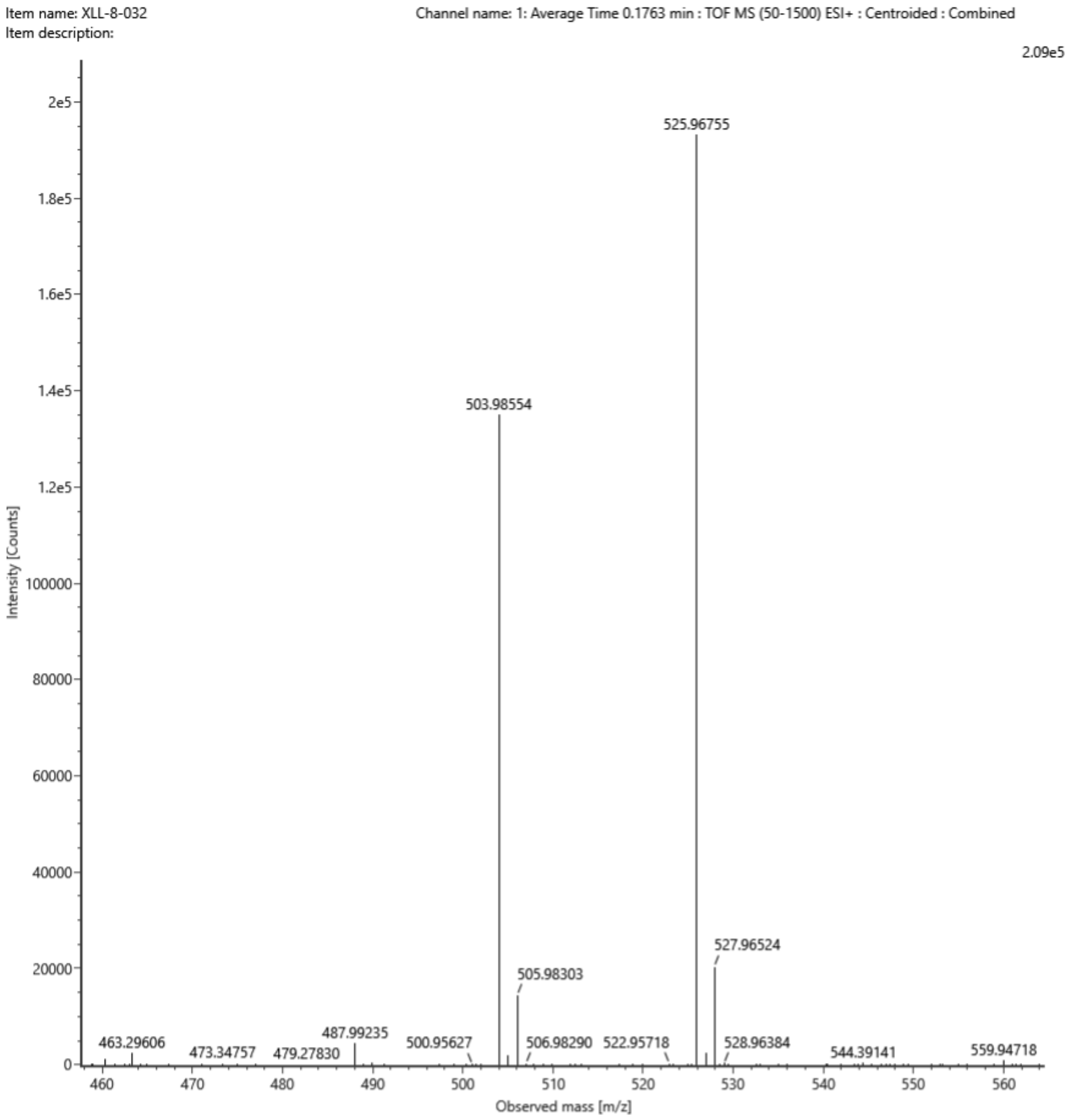

HRMS of compound 7j

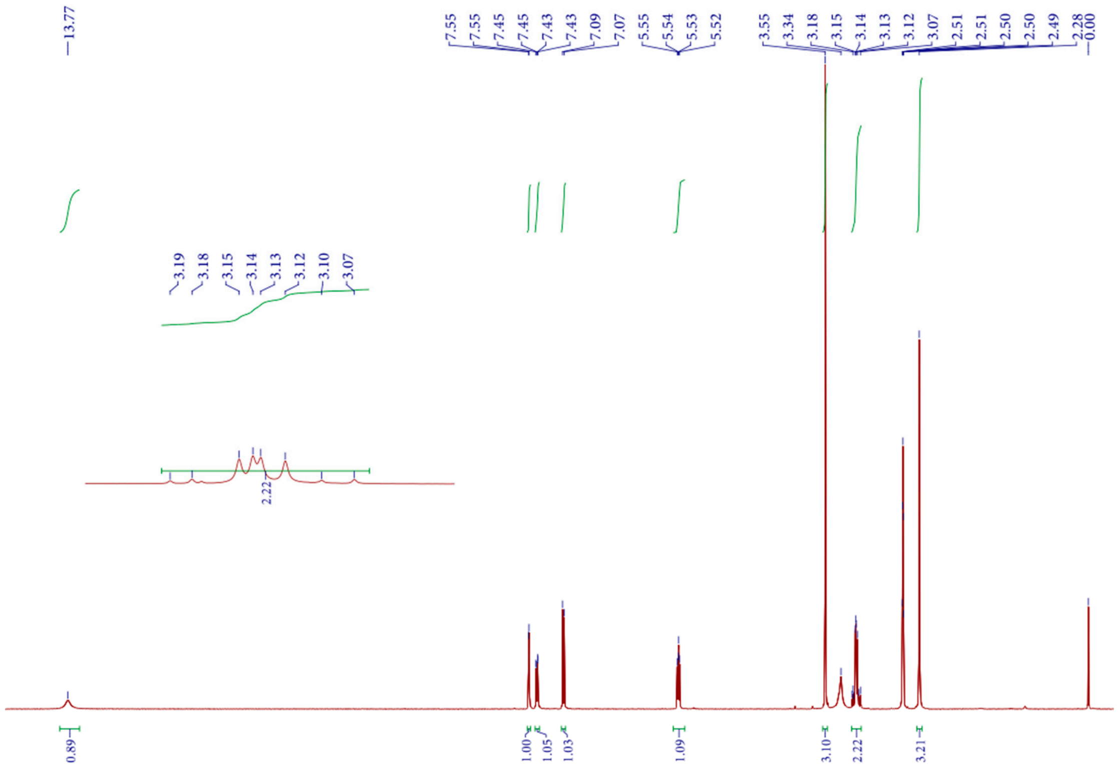

^{1}H NMR of compound 7k

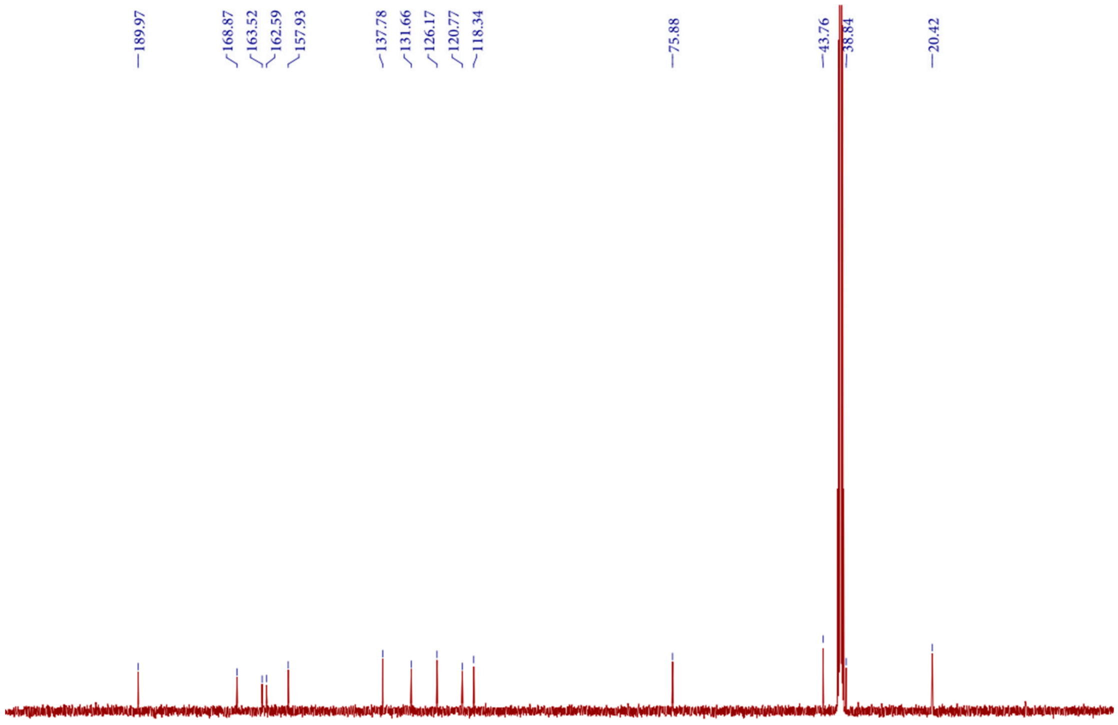

^{13}C NMR of compound 7k

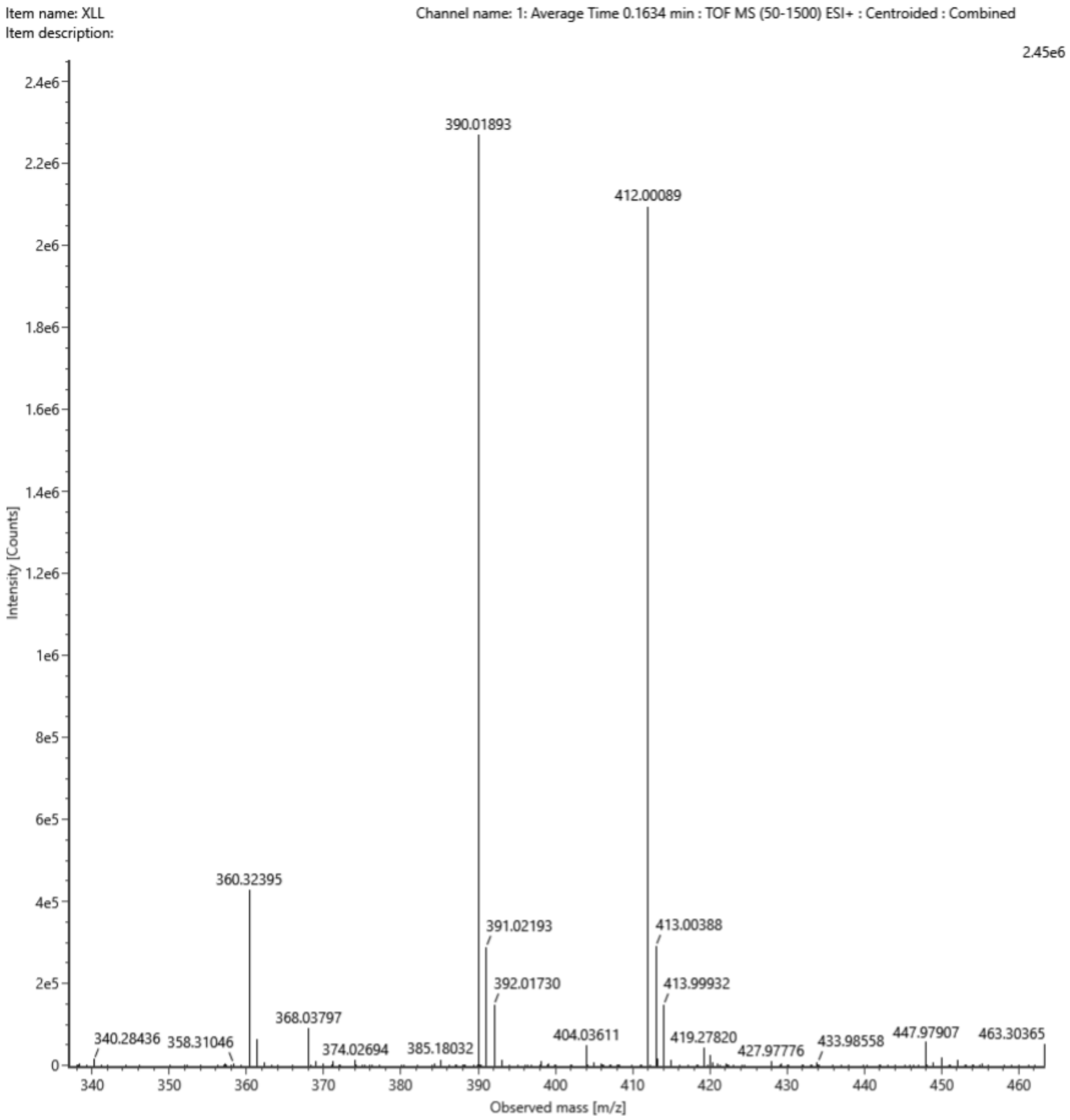

HRMS of compound 7k

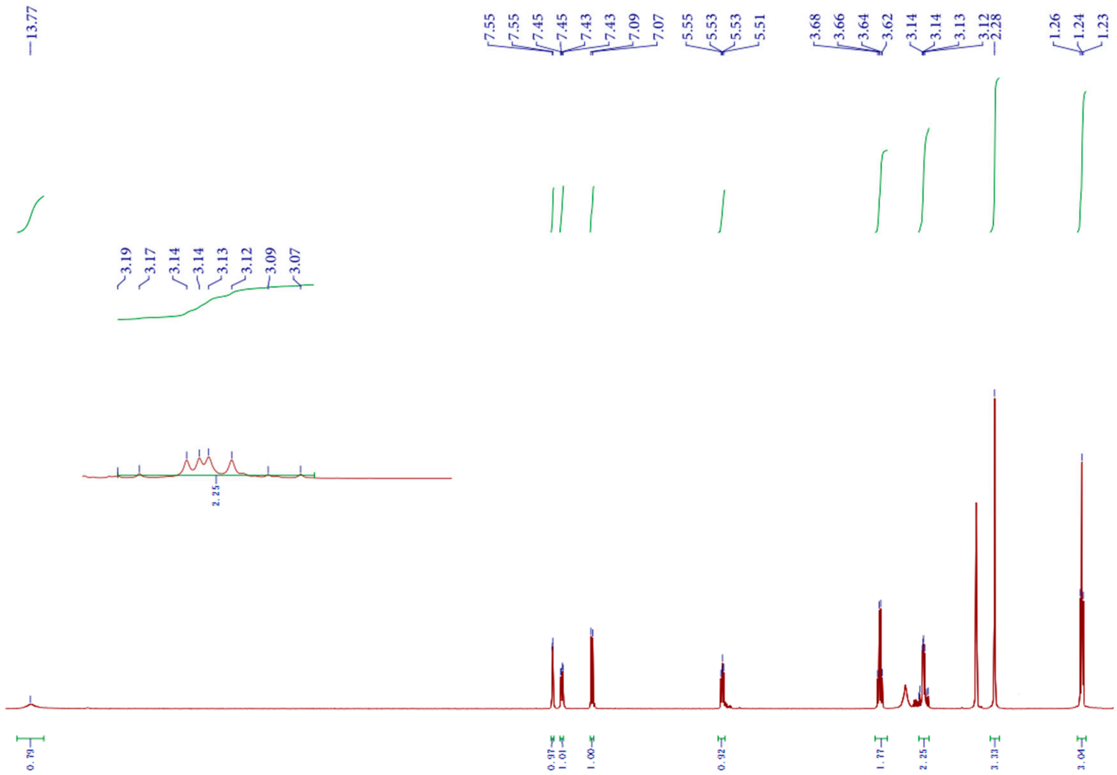

^{1}H NMR of compound 7l

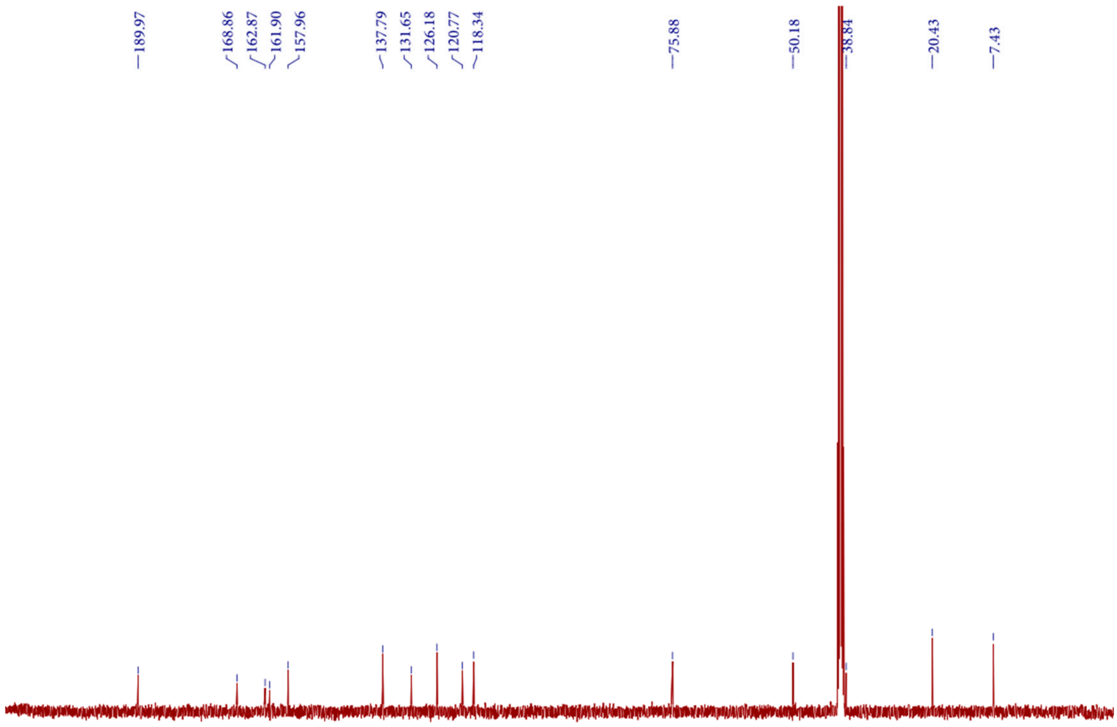

^{13}C NMR of compound 7l

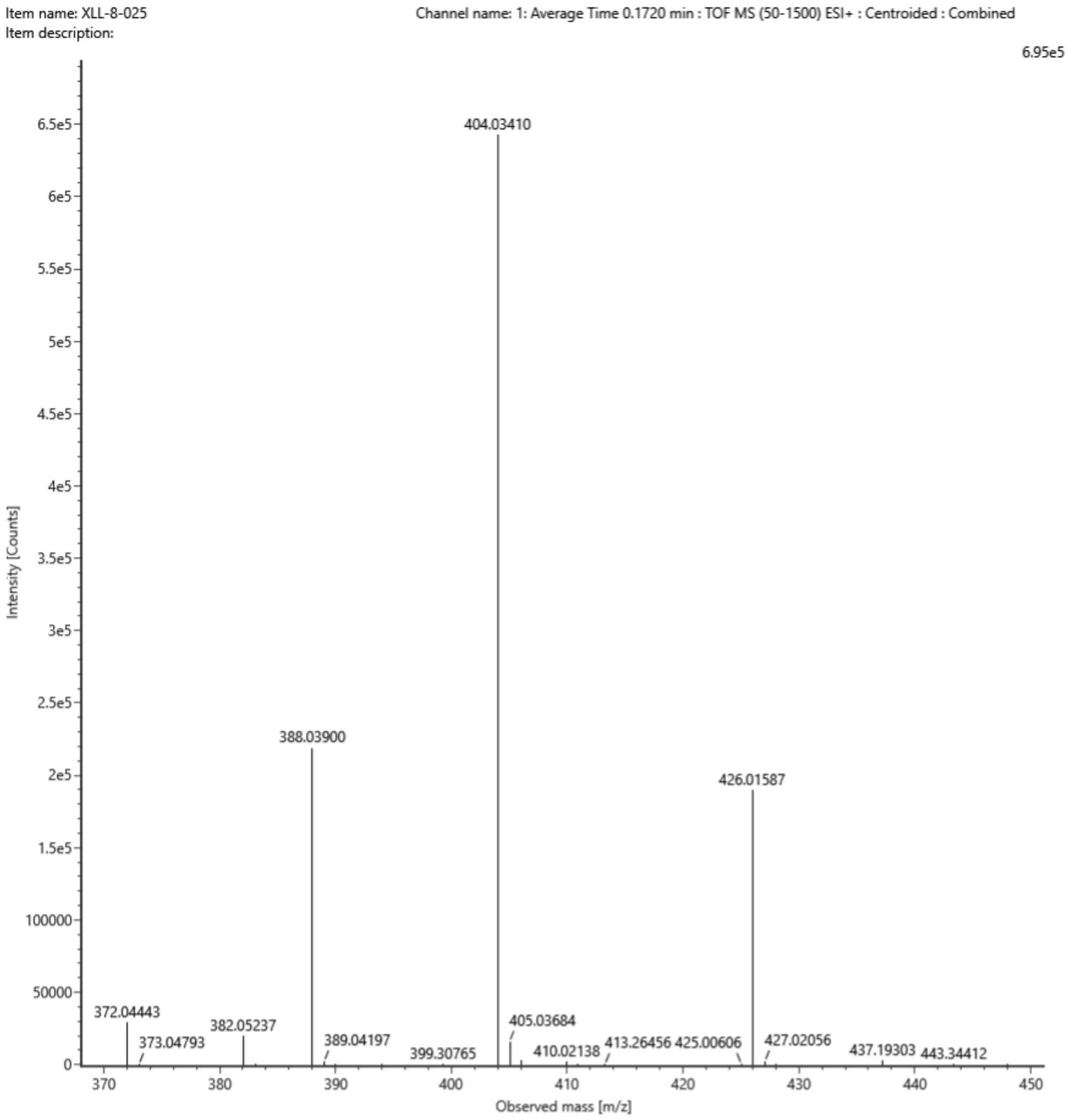

HRMS of compound 7l

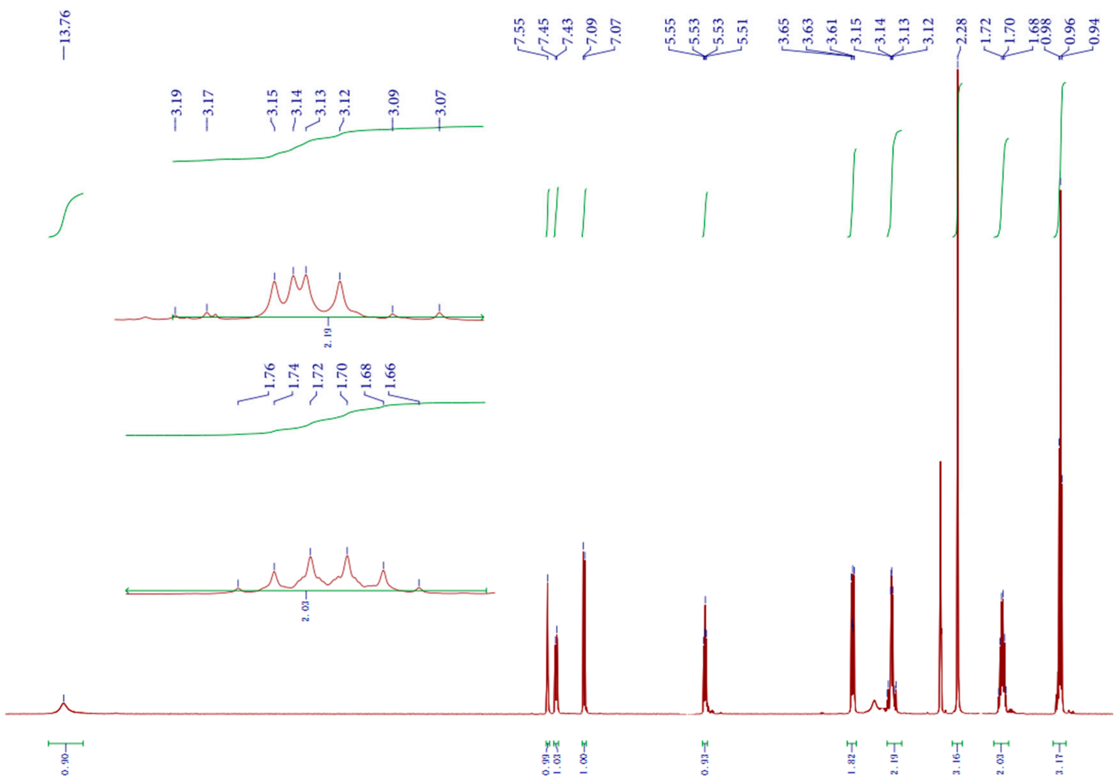

^{1}H NMR of compound 7m

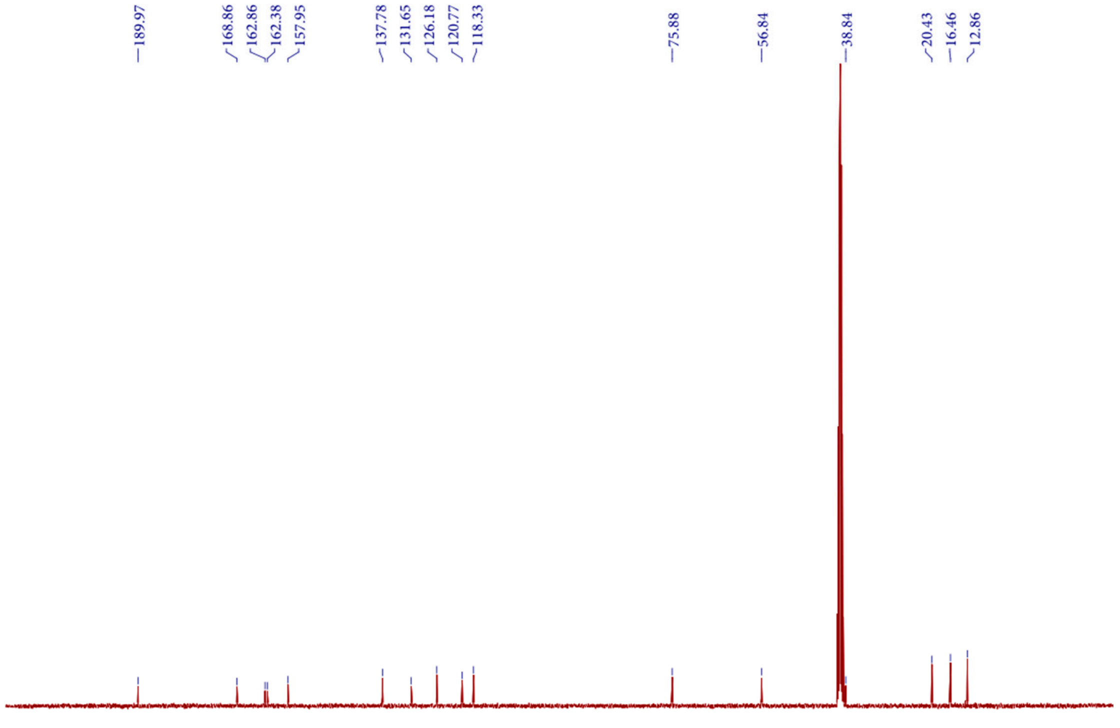

^{13}C NMR of compound 7m

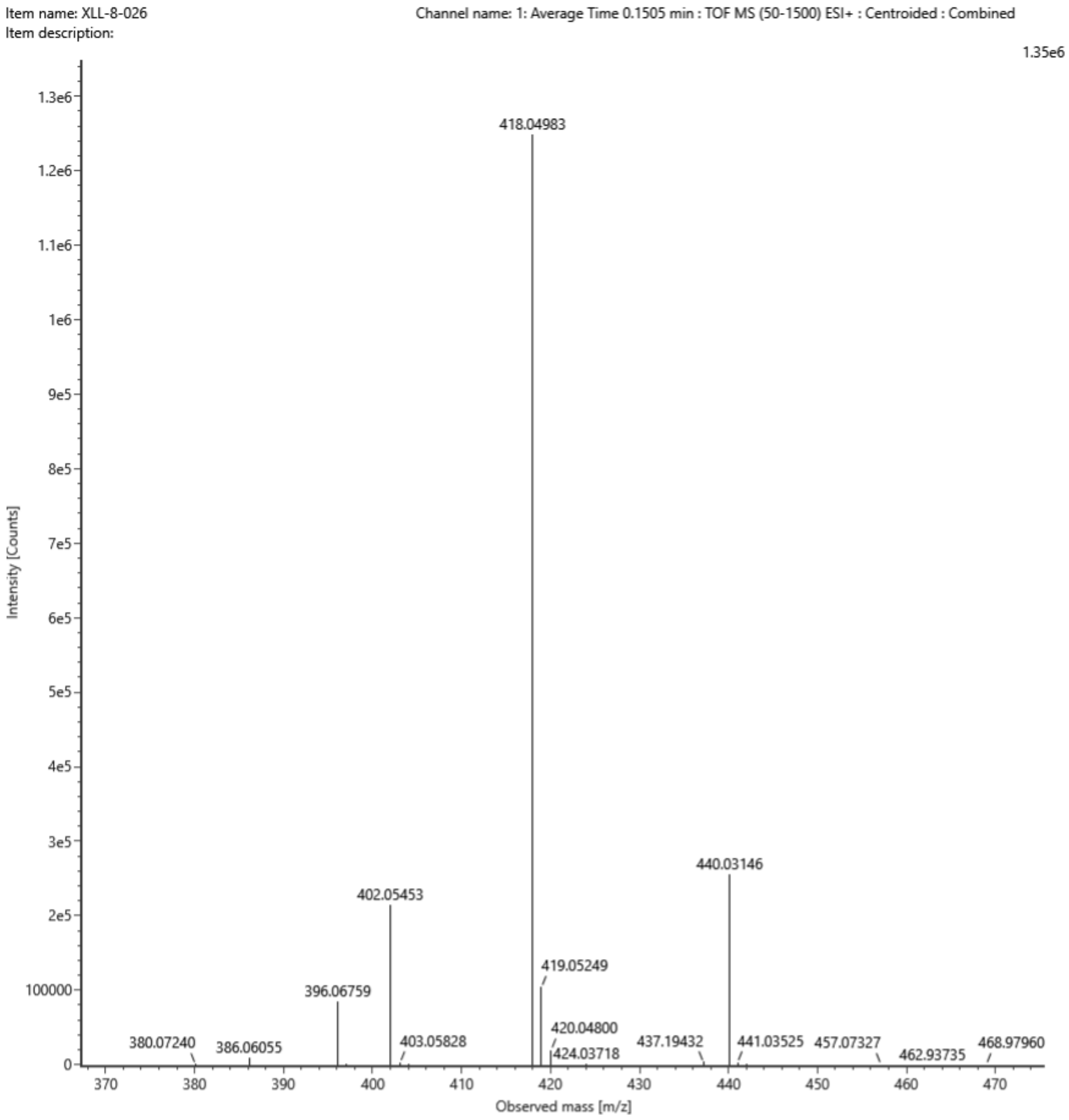

HRMS of compound 7m

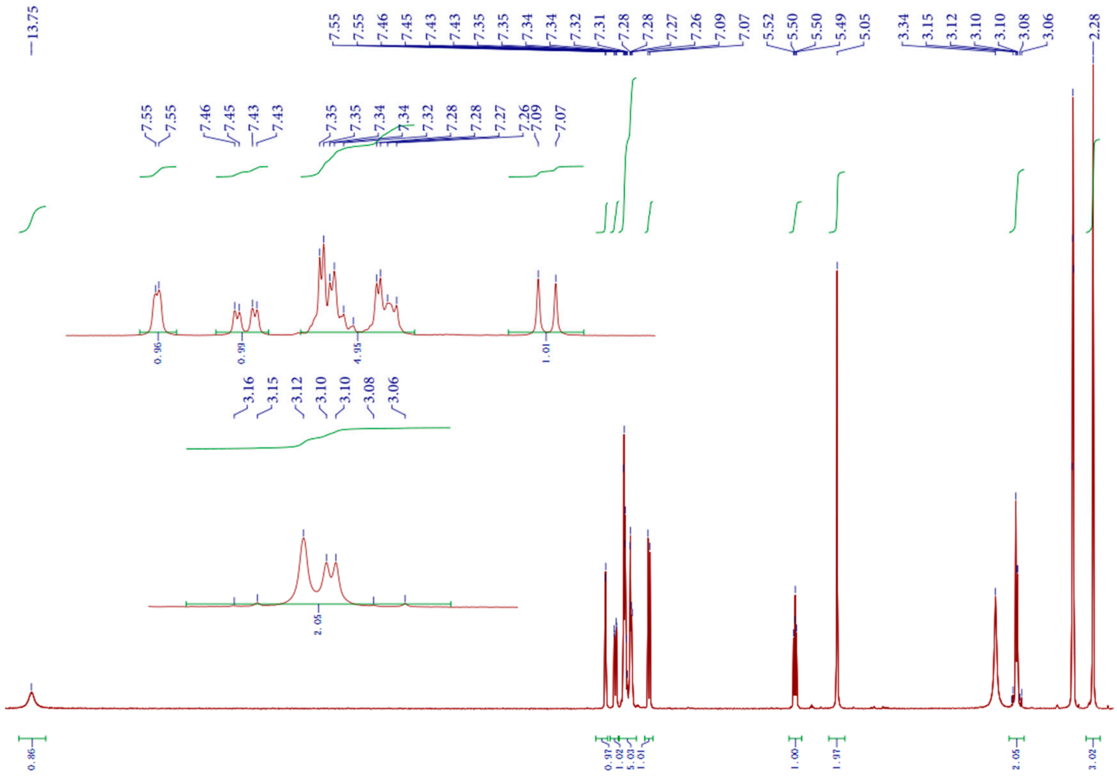

^{1}H NMR of compound 7n

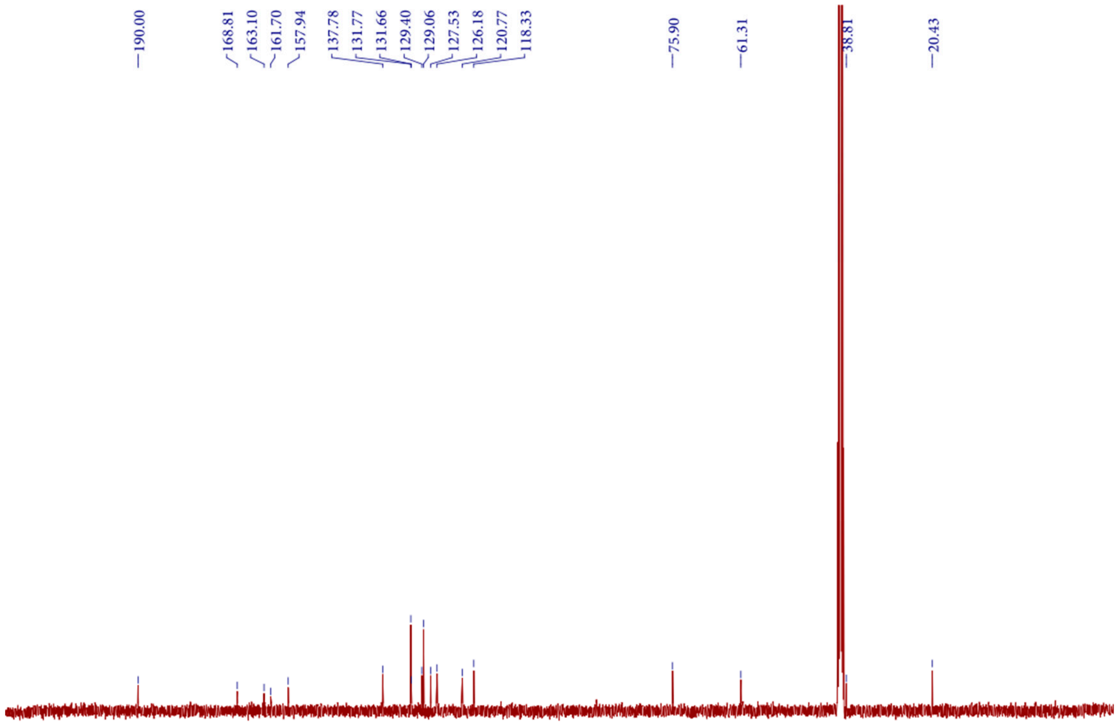

^{13}C NMR of compound 7n

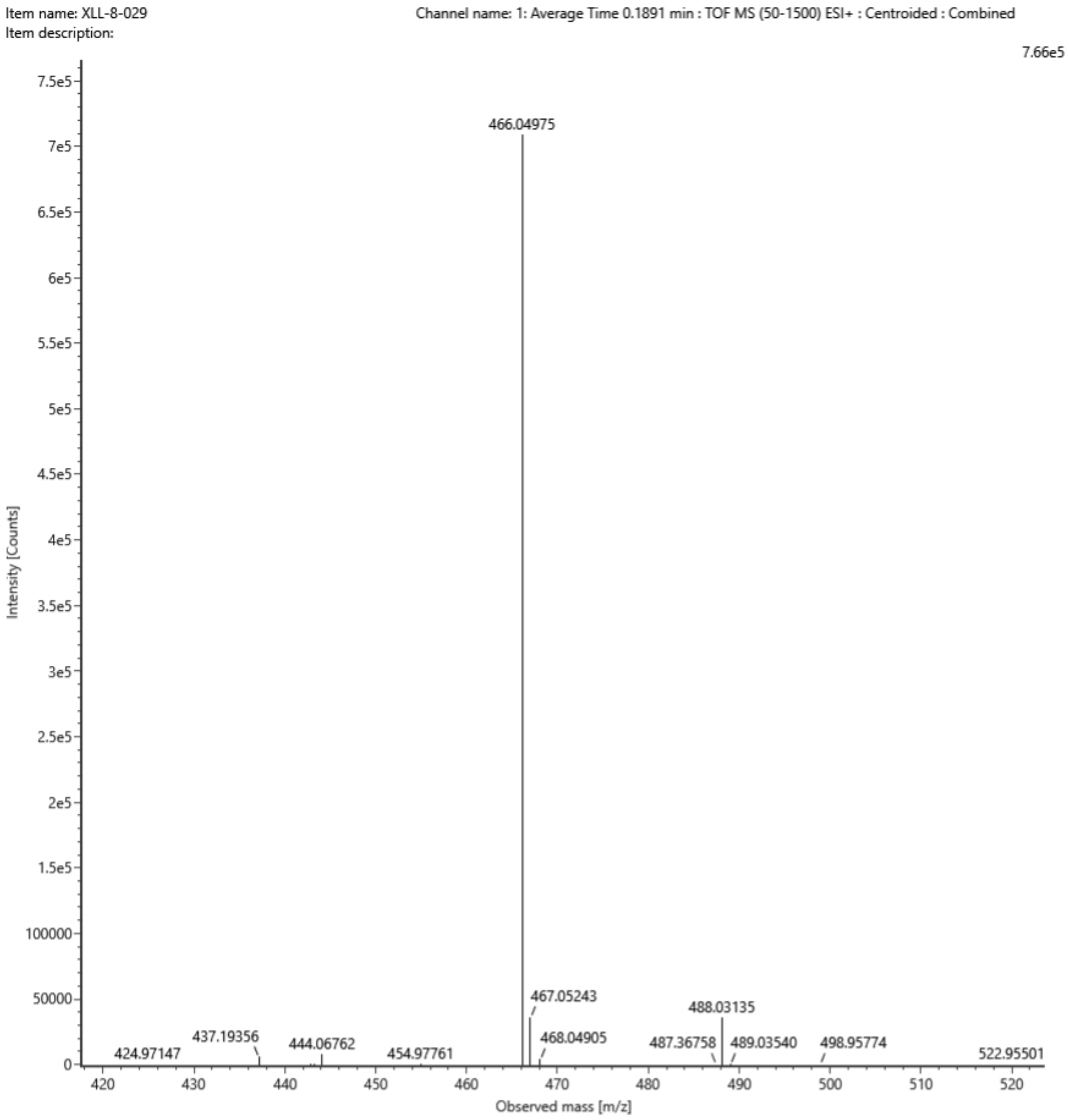

HRMS of compound 7n

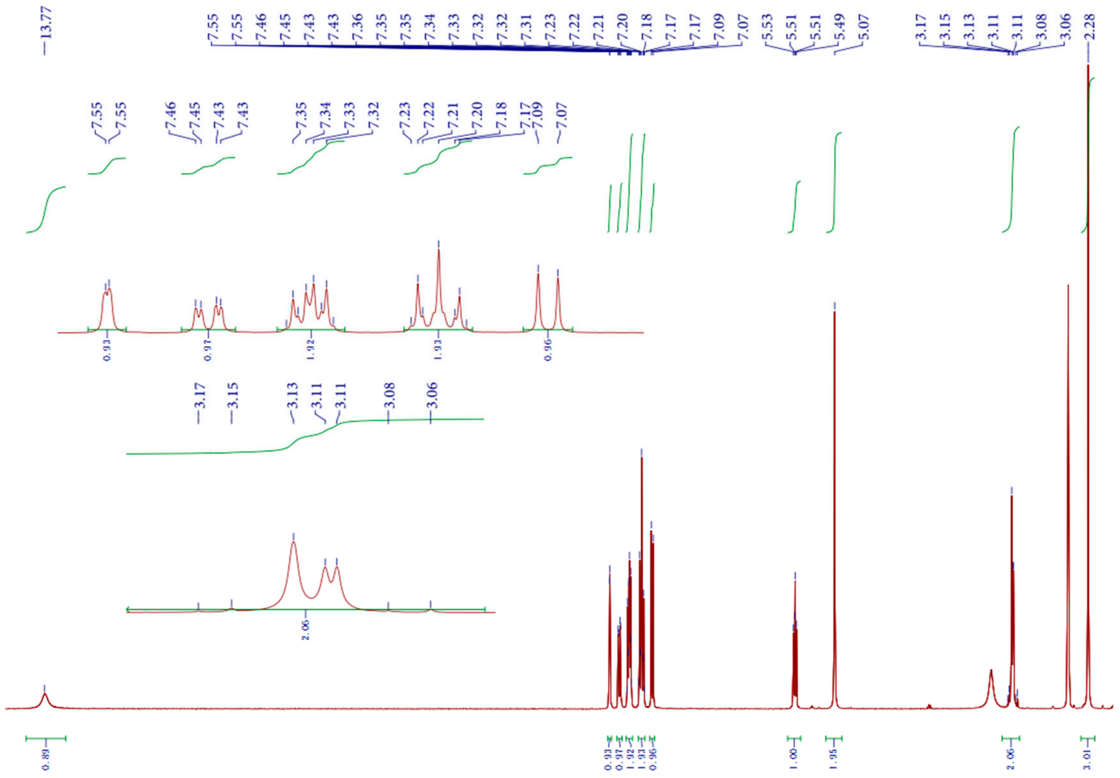

^{1}H NMR of compound 7o

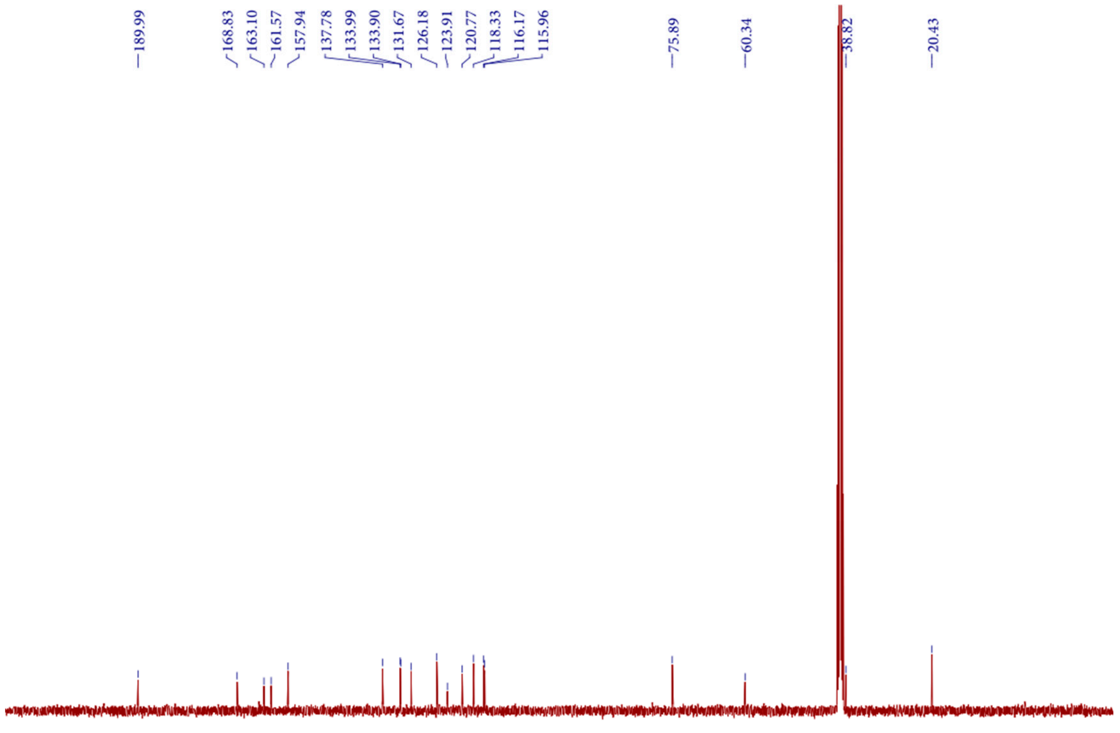

^{13}C NMR of compound 7o

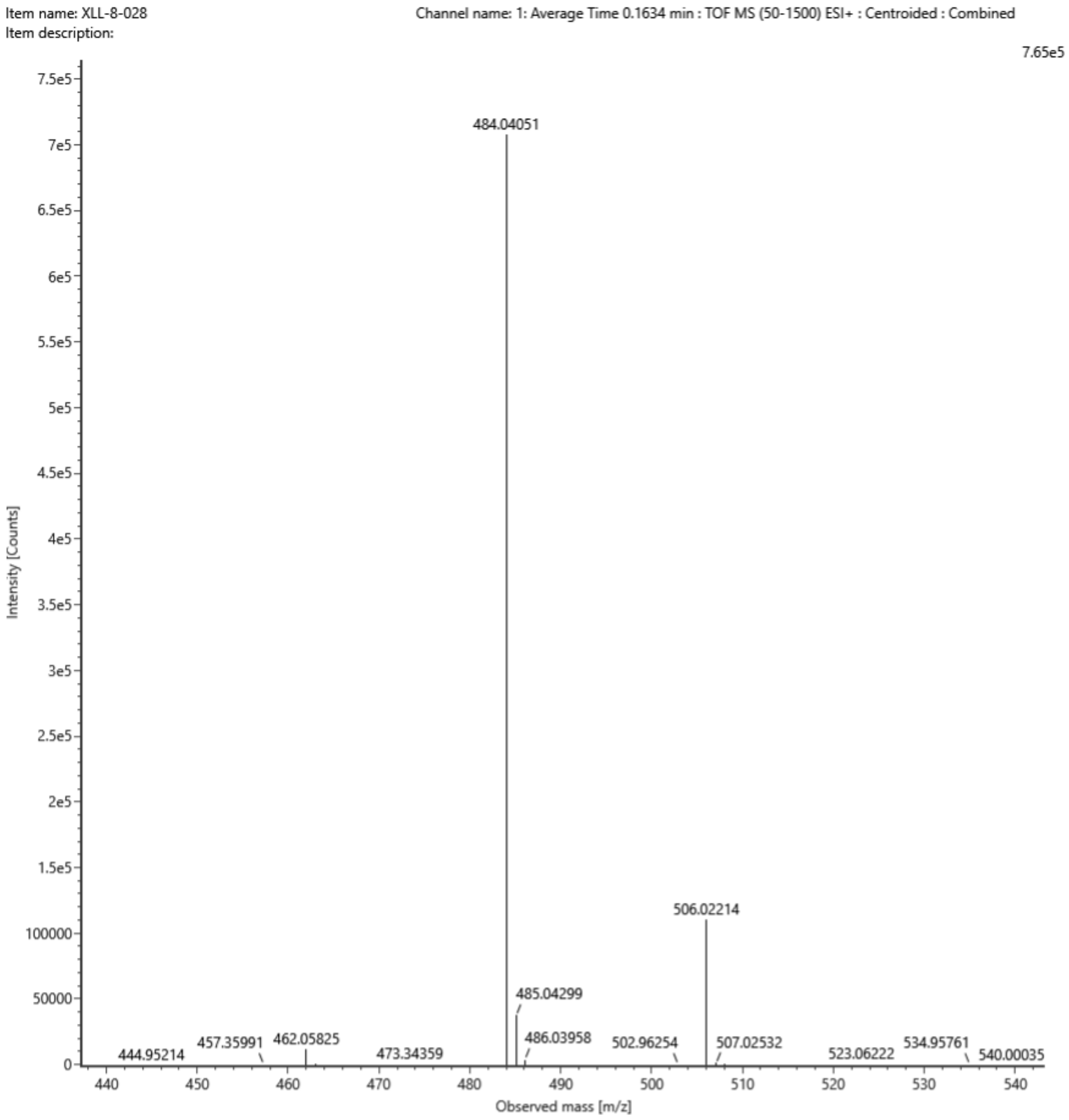

HRMS of compound 7o
